# Light-induced ultrafast spin transport in multilayer metallic films originates from *sp*-*d* spin exchange coupling

**DOI:** 10.1126/sciadv.adi1618

**Published:** 2023-12-15

**Authors:** Zhanghui Chen, Jun-Wei Luo, Lin-Wang Wang

**Affiliations:** ^1^ Institute of Semiconductors, Chinese Academy of Sciences, P.O. Box 912, Beijing 100083, China.; ^2^ Materials Sciences Division, Lawrence Berkeley National Laboratory, One Cyclotron Road, Mail Stop 50F, Berkeley, CA 94720, USA.; ^3^ University of Chinese Academy of Sciences, No.1 Yanqihu East Rd, Huairou District, Beijing 101408, China.

## Abstract

Ultrafast interaction between the femtosecond laser pulse and the magnetic metal provides an efficient way to manipulate the magnetic states of matter. Numerous experimental advancements have been made on multilayer metallic films in the last two decades. However, the underlying physics remains unclear. Here, relying on an efficient ab initio spin dynamics simulation algorithm, we revealed the physics that can unify the progress in different experiments. We found that light-induced ultrafast spin transport in multilayer metallic films originates from the *sp*-*d* spin-exchange interaction, which can induce an ultrafast, large, and pure spin current from ferromagnetic metal to nonmagnetic metal without charge carrier transport. The resulting trends of spin demagnetization and spin flow are consistent with most experiments. It can explain a variety of ultrafast light-spin manipulation experiments with different systems and different pump-probe technologies, covering a wide range of work in this field.

## Teaser

Ab initio spin dynamics simulations reveal the mechanism of laser-controlled magnetism.

## INTRODUCTION

Light-matter interaction provides an efficient approach to manipulate the state of matter (*1*–*8*). In particular, the interaction between the femtosecond laser pulse and the magnetic metal can induce ultrafast demagnetization on the subpicosecond timescale (*1*–*3*, *9*–*25*), orders of magnitude faster than traditional manipulations. Such ultrafast optical control of magnetic states renders it a promising technology for spintronic devices. Despite tremendous experimental progress in this field (*2*, *3*, *11*, *12*, *14*–*20*, *22*, *26*–*29*), the fundamental physics remains unclear, and there are long-standing debates over the dominant mechanisms for the ultrafast demagnetization process (*10*, *20*, *21*, *29*–*42*). A variety of different mechanisms have been proposed in the last 20 years. Overall, there are two types of pictures. One is the spin dissipation picture (*2*, *9*, *12*, *15*, *33*, *43*–*46*), where the total spin angular momentum is changed and dissipated into the reservoirs of other degrees of freedom (e.g., light, lattice, or electronic orbital). Intense debates focus on which reservoir plays the primary role (*2*, *9*, *12*, *15*, *33*, *43*–*45*, *47*, *48*). The other is the carrier-induced spin transport picture (*3*, *14*, *22*, *32*, *49*–*55*), where the total spin angular momentum of the whole system is unchanged, and instead the excited carriers with a polarized spin component are transported from the excited region to the nearby region.

**Fig. 1.**
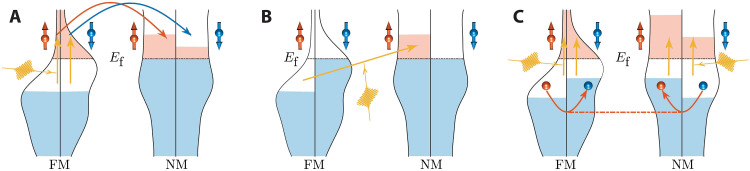
Schematics for different spin transport mechanisms. (**A**) Superdiffusion between the ferromagnetic metal (FM) layer and the nearby nonmagnetic metal (NM) layer. (**B**) Spin-selective charge transfer by OISTR. (**C**) *sp*-*d* spin exchange coupling. The shapes of the density of states (DOS) of FM and NM are taken from the simplification of the DOS curves of Ni and Al, respectively. *E*_f_ denotes the Fermi level. The blue and red colored regions indicate the occupied states in conduction bands and valence bands, respectively, while the white regions indicate the unoccupied states. The yellow wavelets and yellow arrows denote the laser and laser excitation, respectively. The red and blue arrows in (A) indicate the superdiffusive transport of spin-up and spin-down carriers, respectively. The red arrows in (C) indicate how spin exchanges between *d* states of FM and *sp* states of NM. See further descriptions in the text.

There are two well-known spin transport mechanisms. The first is the superdiffusion mechanism (Fig. 1A) (*14*, *32*, *49*–*51*), in which optically excited spin-polarized electrons in the ferromagnetic metal (FM) diffuse into the nearby nonmagnetic metal (NM). Because spin-up and spin-down electrons have different excitation lifetimes and mean free paths, they have different diffusion lengths in NM. This distinction will lead to the transport of a net spin and the depletion of magnetization in FM. The second is spin-selective charge transfer across the FM/NM interface via a mechanism named “optically induced intersite spin transfer (OISTR)” (Fig. 1B) (*3*, *22*, *52*–*54*). In OISTR, the spin-polarized electrons of FM below the Fermi level can be directly excited across the FM/NM interface into the same spin channel of NM above the Fermi level by light. Both the superdiffusion mechanism and the OISTR mechanism require a charge carrier flow from FM to NM that leads to the spin flow. They have been used in the explanation of many different experiments, but their validity has also been hotly debated (*17*, *37*, *56*, *57*). For example, a few experiments have demonstrated that the lifetimes of spin-up carriers and spin-down carriers are almost identical (*17*, *58*), which casts doubt on the fundamental assumptions of very different carrier lifetimes in the superdiffusion model. OISTR, relying on light excitation of FM, is unable to explain many spin transport experiments where the light is shone on the NM side and does not penetrate into the FM side (*32*, *59*). These works indicate the limitations of existing theories.

Here, relying on a highly efficient algorithm for noncollinear time-dependent density functional theory, we remarkably speed up ab initio spin dynamics simulations. This enables us to take real-time snapshots of the spin transport process in relatively thick films and to reexamine the physics behind different experiments. We found that light-induced ultrafast spin transport in multilayer metallic films originates from the spin exchange interaction between localized *d* states of FM and itinerant *sp* states of NM (Fig. 1C). This *sp*-*d* spin exchange mechanism is also free from the shortcomings of the mechanisms mentioned above and can unify the progress in different experiments. We found that the femtosecond laser irradiation induces the carrier excitation (electrons and holes) in FM, NM, or both. The excited carriers in FM and NM exchange their spins rapidly by *sp*-*d* spin-flip exchange coupling (Fig. 1C). This produces an ultrafast, large, and pure spin current from FM to NM without the accompanying charge current (no real particle exchange), and the charge particles no longer serve as the vehicle of spin across the interface. This *sp*-*d* spin exchange is like the exchange interaction between magnetic *d* states and nonmagnetic *sp* states in dilute magnetic semiconductors or alloys (*60*, *61*), but here it occurs at the FM/NM heterostructures and leads to the pure spin transport from FM to NM. Such pure spin transport dominates the spin dynamics of multilayer metallic films. The resulting trends of spin demagnetization and spin flow are consistent with most experimental observations. Through systematic comparisons with experiments, we demonstrated that it can explain a variety of ultrafast light-spin manipulation experiments with different systems (e.g., Ni/Al, Ni/Pt, Au/Co-Pt, Au/Ni, and Fe/Au) and different pump-probe technologies (e.g., pumping of different regions), covering a major range of work in this field. This provides a unified insight into the light-induced spin transport physics as well as the demagnetization amplitude mismatch between theory and experiment in multilayer metallic films.

## RESULTS

One obstacle in previous theoretical studies for ultrafast spin dynamics of complicated systems is the lack of efficient real-time ab initio simulation tools. This has posed a problem for fully understanding the physics of photon-induced spin dynamics in different experiments and is one of the reasons for the long-standing debates. Here, our simulations are based on the noncollinear extension of real-time time-dependent density functional theory (RT-TDDFT; different from the common linear-response TDDFT) (*31*, *62*, *63*). In contrast to many analytical models, RT-TDDFT does not presuppose the physics that might dominate the spin dynamics, nor does it use some presupposed parameters (e.g., carrier lifetimes) that can substantially change the dynamics. For example, we do not presuppose the role of carrier superdiffusion, OISTR, or *sp*-*d* spin exchange. The RT-TDDFT Hamiltonian does not even have an explicit *sp*-*d* spin-flip scattering term. This is unlike the treatment of many traditional approaches, e.g., the well-known Elliott-Yafet electron-electron scattering mechanism (*64*), which explicitly writes down the scattering term (see the Supplementary Materials for further comparisons). Unfortunately, one major disadvantage of RT-TDDFT is its time-consuming nature, which limits its role in spin dynamics simulations of complicated systems.

To well reproduce the light-spin manipulation experiments in multilayer films, we have developed a highly efficient algorithm to do the time evolution based on the adiabatic state basis set and leap-frog iteration (see Methods for details), which is hundreds of times faster than traditional algorithms. This algorithm enables us to directly study the full phenomenon of ultrafast spin dynamics and to simulate relatively thicker films than previously studied, which is particularly important for multilayer systems. The Hamiltonian of our RT-TDDFT code includes all the effects of noncollinear magnetic moments, light-spin, light-orbital, spin-orbit, electron-electron, and electron-phonon interactions. This is an advantage over many previous analytical model studies where some effects (e.g., electron-electron interaction) are difficult to include or to include together. Our RT-TDDFT method also allows us to turn on or off some of these effects, thus studying their roles separately. These features enable us to study all the possible channels for spin evolution and to explore the physics behind different experiments.

Here, we mainly study the Ni thin film (*3*, *13*, *16*, *18*–*20*, *31*–*33*, *37*, *39*, *49*–*51*), one of the most studied systems for ultrafast spin dynamics. We first examine an isolated four-monolayer Ni slab (denoted as Ni_4_). Then, we pack it on top of the Al substrate along the (001) surface. The spin dynamics of the Ni/Al system has been studied by a few ultrafast experiments (*32*, *50*) and classical superdiffusive spin current theories (*49*). We can directly compare our results with these works. Moreover, Al only has *sp* electrons, and *sp*-*d* coupling exists between Ni and Al. This makes it easier for us to identify that the spin exchange between FM and NM (if it exists) is due to the *sp*-*d* exchange coupling (Fig. 1C) rather than the exchange interaction between local magnetic moments (*65*, *66*). To compare with the experiments, we gradually increase Al thickness and consider the systems with 4-, 6-, 8-, and 12-monolayer Al (denoted as Ni_4_Al_4_, Ni_4_Al_6_, Ni_4_Al_8_, and Ni_4_Al_12_, respectively). The spin dynamics trend from thinner to thicker Al will also help in determining the dominant mechanism, as different mechanisms have different dependencies on the substrate thickness. The Ni_4_Al_12_ slab is about 2.7 nm in thickness, longer than ever studied before. These slabs are irradiated by a short, intense, and linearly polarized laser pulse with 600-nm wavelength, 20-fs duration (from 0 to 20 fs, the time between the start and the end of the laser pulse), and 20 mJ/cm^2^ fluence (see the Supplementary Materials for details). To demonstrate the generality of the *sp*-*d* spin transport theory, we will further study the dependence on Ni film thickness and laser parameters later. Several other systems (Ni/Pt and Fe/Au), which have been widely studied experimentally, will also be investigated. After that, we will make a systematic comparison with a series of important experiments in this field.

**Fig. 2.**
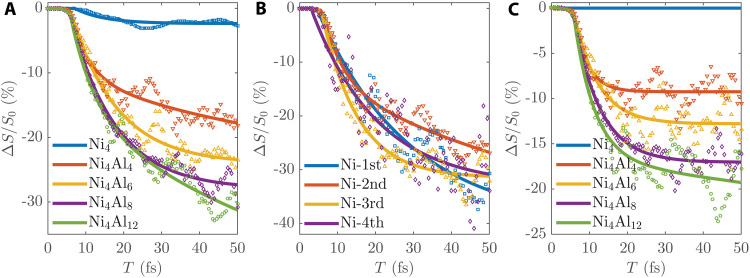
Ni spin evolution in different slabs. (**A**) Demagnetization rates of the whole Ni layers in the original simulations. (**B**) Demagnetization rates of different Ni monolayers of Ni_4_Al_12_ in the original simulations, where Ni-1st is the surface monolayer and Ni-4th is the interface monolayer. (**C**) Demagnetization rates of the whole Ni layers in the simulations with SOC turned off. Δ*S* is the spin change amplitude, and *S*_0_ is the initial spin. Δ*S*/*S*_0_ indicates the demagnetization rate of Ni spin. Lines are the smooth fitting of data points from RT-TDDFT simulations (see the Supplementary Materials).

Figure 2A shows the Ni spin demagnetization of the isolated Ni slab and Ni/Al slabs under laser irradiation. We can see that there is only about 3% spin quenching for the isolated Ni slab (Ni_4_) at 50 fs. This is much smaller than the experimental observations (>30%) in Ni thin films (*3*, *13*, *16*, *18*–*20*, *32*, *33*), a well-known mismatch problem between theory and experiment that has puzzled the field for many years (see more descriptions in the Supplementary Materials). We show here that this rate can be greatly increased with the inclusion of a nearby Al substrate. It can go up to about 17% in the thin Ni_4_Al_4_ slab under the same light excitation. It goes higher with the increase of Al thickness and exceeds 30% in the Ni_4_Al_12_ slab. This is 10 times the rate for the Ni_4_ slab and already comparable to the experimental values. As seen from the trend from Ni_4_Al_4_ to Ni_4_Al_12_, the quenching rate can be even higher in a thicker Al layer. This indicates that our RT-TDDFT simulations have well reproduced the experimental spin quenching results on FM/NM multilayer thin films on the femtosecond timescale. To see whether this speedup works only for the interface layer, we calculated the quenching rate for each Ni layer. Figure 2B shows the results. We can see that the large spin quenching occurs not only around the interface but also in the whole Ni film. These results demonstrate that a nearby NM layer dramatically accelerates the spin quenching of FM thin films, which is in agreement with many experiments for the comparison between FM/NM, FM bulk, and FM/insulator systems (these experiments will be discussed later) (*3*, *67*).

As mentioned above, the spin quenching of FM arises from two different ways: spin dissipation and spin transport. To distinguish their roles, we removed the spin-orbit coupling (SOC) term from the Hamiltonian and ran the simulations with the same excitation for these systems. By doing so, we turned off the spin dissipation through the SOC channel. Because the SOC channel is necessary for spin angular momentum to dissipate to other degrees of freedom (*38*, *45*), this will completely remove the spin dissipation contribution, and the total spin of the whole system will be conserved. Figure 2C shows the corresponding results. As we can see, the spin of the Ni_4_ slab remains unchanged throughout the dynamics because there is no channel for both dissipation and transport. With the presence of the Al layer, strong spin decay emerges with time, and this decay is fully contributed by the spin transport to the nearby Al layer. The magnitude of such spin transport also increases with the thickness of the Al layer. The Ni quenching rate reaches about 20% in Ni_4_Al_12_, which is about two-thirds of the total quenching in Fig. 2A. This demonstrates the dominant role of spin transport in demagnetization. By comparing Fig. 2A and Fig. 2C, we can also deduce the contribution from SOC-induced spin dissipation. For example, the spin quenching rate from dissipation is about 10% in Ni_4_Al_12_. One interesting finding is that this value is higher than the 3% in the isolated Ni_4_ slab. This could be because the spin transport to the Al layer changes the Ni electronic structure, which further changes SOC and spin dissipation.

**Fig. 3.**
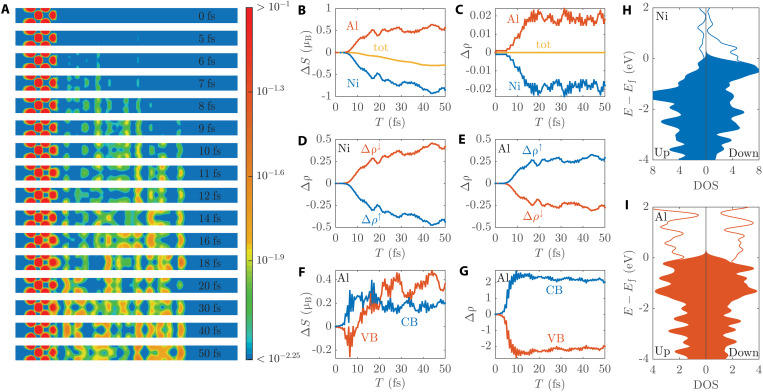
Spin structure and electronic structure evolution of the Ni_4_Al_12_ slab. (**A**) Snapshots of real-space spin density at different times, where the value is indicated by the color bar. (**B** and **C**) Spin change (Δ*S*) and charge change (Δρ) of Ni, Al, and total. (**D** and **E**) Spin-decomposed charge change in Ni and Al. (**F** and **G**) Spin change and charge change in conduction bands (CB) and valence bands (VB) of Al. (**H** and **I**) Spin-decomposed DOS of Ni and Al in the ground state, where *E*_f_ indicates the Fermi level and the shaded regions indicate the occupied states with Gaussian smearing.

To understand the underlying physics for such ultrafast, large, and thickness-dependent spin transport, we further calculated the evolution of spin structures and electronic structures for the longest Ni_4_Al_12_ slab. Figure 3A shows the snapshots of real-space spin density at different times. It can be seen that the spin initially stays in the Ni layer. With laser irradiation, there is an obvious spin transport from Ni to Al. The spin moves quickly across the interface and reaches the Al surface. At the end, it spreads nearly uniformly in the Al layer. This indicates that the eventual spin transport is long range and the whole Al layer serves as a reservoir to receive the injected angular momentum. This could explain to some extent why a larger spin transport is observed in a thicker Al layer (Fig. 2C). Figure 3B shows the quantitative spin changes of Fig. 3A in Ni, Al, and the whole system. We can see that Ni spin decreases and meanwhile Al spin increases. The curve of Ni in Fig. 3B is the point data in Fig. 2A without dividing the initial spin *S*_0_, and its change is contributed by both dissipation and transport as discussed earlier. Different from Ni, the SOC in Al is much weaker, and its dissipation channel is negligible. Thus, the Al spin change is fully contributed by the injection from Ni. Its sharp rise is the result of large spin transport. As for the total spin of the whole system, it decays with time (Fig. 3B). Since spin transport conserves the total angular momentum, the total spin decay results only from spin dissipation and mainly from the SOC channel of Ni.

Having been consistent with experiments and identified the role of spin transport, the next question is: What is the mechanism for this spin transport? In previous works, the ultrafast spin transport between FM and NM is usually explained by the superdiffusion mechanism (Fig. 1A) or the OISTR mechanism (Fig. 1B). Both of them rely on the charge particle flow as the carrier of spin across the interface. We should observe a strong correlation between charge injection and spin injection if either of them is dominant. Take (*3*) as an example. With OISTR, it is assumed that electrons and spins are injected from Ni into the nearby Pt layer at the same time. To check this, we calculated the local charge change in Ni and Al, as shown in Fig. 3C. It can be seen that the charge flow from Ni to Al is very tiny, about 25 times smaller than the spin flow counterpart. We further analyzed the distribution of this tiny injected charge particle on each Al monolayer (see fig. S6). We found that the charge injection mostly occurs around the interface and does not penetrate deeply into the Al film, which is different from the long-range spin flow (Fig. 3A and fig. S5). The charge loss in Ni also mostly occurs at the interface Ni layer (fig. S6), different from the spin loss in the whole Ni film as shown in Fig. 2B. Moreover, contrary to the thickness dependence of spin injection (Fig. 2C), the amount of charge injection does not increase with Al film thickness and there is no obvious difference from Ni_4_Al_4_ to Ni_4_Al_12_ (see fig. S7). These results are more like an interface local charge change effect after laser excitation. Such a tiny and localized charge change cannot be the reason for the large and long-range spin flow, and cannot explain the spin-thickness relation (Fig. 2C). These results cast some doubt on the superdiffusion mechanism and the OISTR mechanism for our simulations.

Figure 3 (D and E) further shows the change of local charge with different spin components. It can be seen that spin-up particles transfer to spin-down particles in Ni, and in the meantime, Al has the inverse transfer from spin-down to spin-up. This is the spin exchange process described in Fig. 1C. Thus, it is the *sp*-*d* spin exchange coupling that causes the large pure spin transport here. Note that the spin-decomposed Δρ in Fig. 3D is bigger than in Fig. 3E. This is because Ni has an additional spin dissipation channel for the transfer from spin up to spin down. If we turn off SOC, spin-decomposed Δρ in Fig. 3D will be the same as in Fig. 3E (see fig. S8), demonstrating an exact spin-exchange process between Ni and Al.

It should be noted that the superdiffusion model is semiclassical and is typically used to simulate thick systems and long timescales (e.g., much longer than the spin diffusion lengths and spin lifetimes of spin-up and spin-down carriers). The comparison between superdiffusion and *sp*-*d* spin exchange in this thin system (∼2.7 nm) and short timescale (50 fs) is not fair. Nevertheless, because of their distinct features, it is still valuable to distinguish their charge dynamics and spin dynamics. In most superdiffusion (*49*, *68*) and superdiffusion-like (*69*) simulations, the injection of spin-polarized electrons from FM to NM is assumed to be rapidly counteracted by a screening current of unpolarized electrons from NM to FM. Previous experiments have shown that the charge screening time in Ni is relatively long, about 1.5 fs (*70*) or 2 to 3 fs (*71*). Thus, there should be a time delay between the forward charge flow and the reverse screening charge flow. If spin transport is induced by such spin-polarized charge flow, we should observe a large charge fluctuation or an obvious correlation between spin dynamics and charge dynamics. However, these phenomena do not appear in our simulations. The tiny charge fluctuation mostly occurs at the interface, while the spin transport goes to the far distance (figs. S5 and S6). Delayed and canceled charge flow also cannot explain the different dependencies of charge dynamics and spin dynamics on Al thickness (Fig. 2 and fig. S7). These results do not support the superdiffusion mechanism in small systems and on short timescales. Nevertheless, we still cannot rule it out in large systems and on long timescales based on these data.

As for the OISTR model, it is derived from a close length scale and timescale as our *sp*-*d* spin exchange model. It can also lead to a canceled charge current, resulting in a pure spin current in some systems [e.g., FeNi alloy (*22*)]. To further distinguish OISTR and *sp*-*d* spin exchange, we analyzed the spin evolution at different electronic bands. Note that for the sake of analysis, we use the terms conduction band electrons and valence band holes to describe the carriers above and below the Fermi level, respectively. Figure 3 (F and G) shows the corresponding results. First, we can see that the injected spins are distributed in both conduction and valence bands of Al (Fig. 3F). There are even more spins in valence band holes. This indicates that spin exchange takes place in both conduction and valence bands. This differs from OISTR (*3*), where the valence band spin of FM is directly injected into the conduction band of NM (Fig. 1B). Second, there is a time delay between electronic excitation and spin transport (Fig. 3, F and G). It can be seen that the Al electronic excitation has reached its maximum at 10 fs (Fig. 3G). On the other hand, the large spin transport only begins at around 10 fs (Fig. 3, A, B, E, and F) and continues even after the laser has disappeared (after 20 fs). This is also different from OISTR, where spin transport and electronic excitation couple together, and should occur simultaneously. Third, OISTR relies on inter-site charge transfer between neighboring atoms and operates on a very short spatial scale. The injected spin should decay rapidly with the distance from the interface, whereas here the spin distributes approximately uniformly after a short time (Fig. 3A and fig. S5). Fourth, the optical excitation of Ni is similar from Ni_4_Al_4_ to Ni_4_Al_12_. The Al conduction band is very empty (Fig. 3I) and can accept large excitations from Ni (Fig. 3H). If OISTR dominates the spin transport, then the amounts of spin injection for these slabs should be similar as well, rather than showing a very clear thickness dependence. In contrast, in the *sp*-*d* spin exchange picture, all the excited *sp* states act as a reservoir to receive the injected spin from *d* states. The thicker Al slab has more excited states and thus a bigger reservoir to receive more spins. All this shows that OISTR is not an appropriate model to describe the spin dynamics here.

To further distinguish these models and gain more insight into spin dynamics, we have added two different types of simulations.

**Fig. 4.**
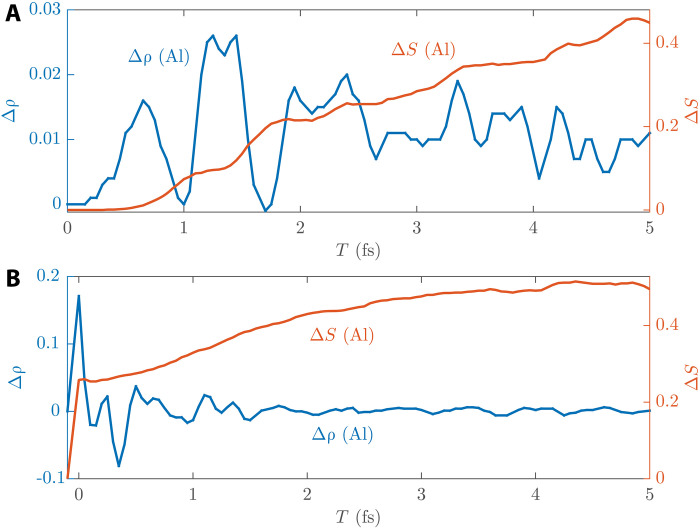
Al dynamics of the Ni_4_Al_12_ slab. (**A**) Laser excitation with 2-fs duration. (**B**) Manual excitation at 0 fs, where the value below 0 fs is the referenced ground state before excitation. The left *y*-axis value is the Al charge change (Δρ), and the right *y*-axis value is the Al spin change (Δ*S*).

First, we excited the Ni_4_Al_12_ system using two very short lasers, one with 2-fs duration (fig. S9, from 0 to 2 fs, close to the charge screening time) and the other with 0.5-fs duration (fig. S11, from 0 to 0.5 fs, shorter than the charge screening time). If charge carrier transport is the dominant mechanism, we should observe an obvious charge screening dynamics that is correlated with spin dynamics. Figure 4A shows the result for 2-fs laser and compares the charge change and spin change of Al. The result for 0.5-fs laser is shown in fig. S11. It can be seen that the charge dynamics amplitude (Δρ) is still much smaller than the spin amplitude (Δ*S*). There is no large forward charge flow and reverse screening flow to correlate with spin flow. This tiny charge fluctuation is more like the adiabatic motion of the whole electron sea due to the fluctuation of the laser electric field. Additionally, in the 2-fs laser case, spin injection from Ni to Al after the laser pulse (i.e., after 2 fs) continues at almost the same rate as during the laser pulse. In the 0.5-fs case, there is almost no spin transport during the laser illumination, and the main spin transport takes place after the laser has disappeared. This shows the effect that *sp*-*d* spin exchange can continue longer after the laser pulse, and indicates the decoupling of optical excitation and spin transport on the timescale, which is very different from OISTR, where spin transport and optical excitation occur simultaneously. Thus, the role of the light pulse is not to excite electrons from FM to NM (i.e., an OISTR process), but is a heating effect that generates more nonequilibrium carriers and increases their temperatures for the subsequent *sp*-*d* spin exchange.

Second, we performed comparable simulations by manually exciting electrons from Ni to Al, and investigated the charge screening dynamics and spin dynamics. This is achieved by manually exciting the electrons from the occupied states whose wave functions are mostly within the Ni region and whose energies are below the Fermi level, to the unoccupied states whose wave functions are mostly within the Al region and whose energies are above the Fermi level. This is also the OISTR process described in the literature. Figure 4B shows the results. As can be seen, there is about 0.17 electron charge and about 0.25 μ_B_ spin injected into Al at 0 fs. The difference between charge injection and spin injection is mainly due to the wave function being noncollinear and delocalized. After this manual excitation (i.e., after 0 fs), there is a large reverse screening flow from Al to Ni, and a following large charge fluctuation that continues until 1.5 to 2 fs. This is in accordance with the charge screening timescale in the literature. Such charge dynamics behavior does not appear in the above simulations with very short lasers. Thus, the laser excitation dynamics is unlike the OISTR process and also unlike the screening process of a large charge flow.

In addition, we can see that the Al spin keeps increasing after the manual excitation and its spin dynamics has no correlation with the charge fluctuation. This is not consistent with either OISTR or superdiffusion. In OISTR, spin injection and optical excitation couple together. After excitation, spin injection should have ended. In the superdiffusion model, spin-polarized charge injection is counteracted by a screening current of unpolarized electrons. Thus, the Al spin should also remain unchanged during the charge screening time and the later time. The continuously increasing spin here is hardly explained by these two models but can be explained by our *sp*-*d* spin exchange model. The manual excitation raises the electron temperature and breaks the initial equilibrium of *sp*-*d* spin exchange, driving the continuous spin exchange after the excitation.

We also performed three more similar simulations with manual excitations coming from different bands and different numbers of electrons. The results are shown in fig. S12, which are very similar to those in Fig. 4B. This indicates that the observed charge screening dynamics and the continuous spin transport are not due to a specific excitation but are a general phenomenon.

These evidences support that the spin transport here is due to the *sp*-*d* spin exchange process after optical excitation of FM and NM. It is a “two-step” process. The light-induced excitation only occurs within each material. However, after the excitation, due to *sp*-*d* exchange coupling between Ni and Al, there is correlated spin flipping inside Ni and Al. The fact that there are excited states in both Ni and Al makes such spin flipping possible. For example, if there is no excited state in Al, then a relatively “closed shell” will make such flipping difficult. In more detail, we can assume a fully occupied valence band. To achieve *sp*-*d* spin exchange between FM and NM, a spin-down state in NM needs to switch to a spin-up state. However, if the valence band is fully occupied, there will be no vacant spin-up state (i.e., no “room”) to accept such a switch. Because Ni and Al are metals, their bands are not fully occupied at room temperature. There are a certain number of thermal equilibrium states near the Fermi level, and these states can be involved in the spin-switching process. With light excitation, there will be many more nonequilibrium excited states (either unoccupied holes or occupied electrons) far below or above the Fermi level. This generates a large number of free carriers with the source spin states (e.g., spin down) to start the spin-switching process, and also generates a large number of empty “rooms” with the target spin states (e.g., spin up) to complete the switch. It is such an *sp*-*d* spin exchange process that causes the observed ultrafast, large, pure, and long-range spin transport from FM to NM. Note that *sp*-*d* spin exchange can also occur when only FM or NM is optically excited because of the existence of delocalized thermal states. We will demonstrate this point later.

From the point of view of scattering, this *sp*-*d* spin exchange coupling is a type of electron-electron spin-flip scattering. It is similar to the well-known spin-flip scattering in ferromagnetic semiconductors [e.g., Mn-doped GaAs and InAs (*60*, *61*)], but here it takes place within a heterostructure consisting of FM and NM, and leads to spin transport from the FM side to the NM side. It is also somewhat like the Ruderman-Kittel-Kasuya-Yosida (RKKY) effect where itinerant states mediate the exchange interaction between localized states, or the Kondo effect where itinerant states and localized states interact locally (*66*, *72*–*76*). This *sp*-*d* spin-flip scattering initially stays at a dynamical equilibrium state. Light excitation (at FM, NM, or both) generates more carriers and also raises the carrier temperatures, which breaks the initial equilibrium and drives the spin transfer from *d* states to *sp* states. Due to the weak wave function overlap, the far-away *d* electrons and *sp* electrons do not couple directly but are mediated by the adjacent *sp* electrons. The *d*-spin is first transferred to the interface *sp* electrons by *sp*-*d* spin exchange, and then to the distant *sp* electrons by *sp*-*sp* spin exchange. Although such indirect coupling is weak at one time, the amount of spin exchange can accumulate over time. This is evident in Fig. 3A (e.g., from 6 to 10 fs), where spin transport from FM-*d* to NM-*sp* initially occurs at the interface and then gradually moves toward the far distance in NM. The relatively long-range and indirect *sp*-*d* exchange coupling has been reported in some previous works. For example, Korenev *et al.* (*77*, *78*) demonstrated a robust exchange coupling between Co-*d* and CdTe-*p* that does not vary with barrier width up to 30 nm. Although the coupling in some of these works is mediated by other types of particles (phonons or magnons), they demonstrate that the effective *sp*-*d* exchange coupling can reach a very long distance with time.

Note that this *sp*-*d* exchange-induced spin-flip scattering is different from the well-known Elliott-Yafet electron-electron Coulomb interaction (*64*), which is widely used in the understanding of ultrafast demagnetization. Elliott-Yafet scattering contributes to the spin dissipation of the whole system. Its driving force for spin dissipation is SOC. In comparison, *sp*-*d* spin exchange does not change the total spin of the whole system, but only redistributes the spin between FM and NM. It also does not depend on SOC. Even if we turn off SOC, there is still strong spin exchange between FM and NM (as shown in Fig. 2C and fig. S8).

To further verify the above picture, we conducted simulations by shining the laser on different regions of the slab. This enables us to control the carrier excitations in different regions, which are critical for *sp*-*d* spin exchange as mentioned above. In the original simulation, we shined the laser on the whole slab. We added two tests by exciting only the Ni region or only the Al region. This is achieved in the TDDFT simulation by applying the electric/magnetic field of the same laser pulse only to a specific region. Such simulations also correspond to many experiments where only FM or NM is optically excited.

**Fig. 5.**
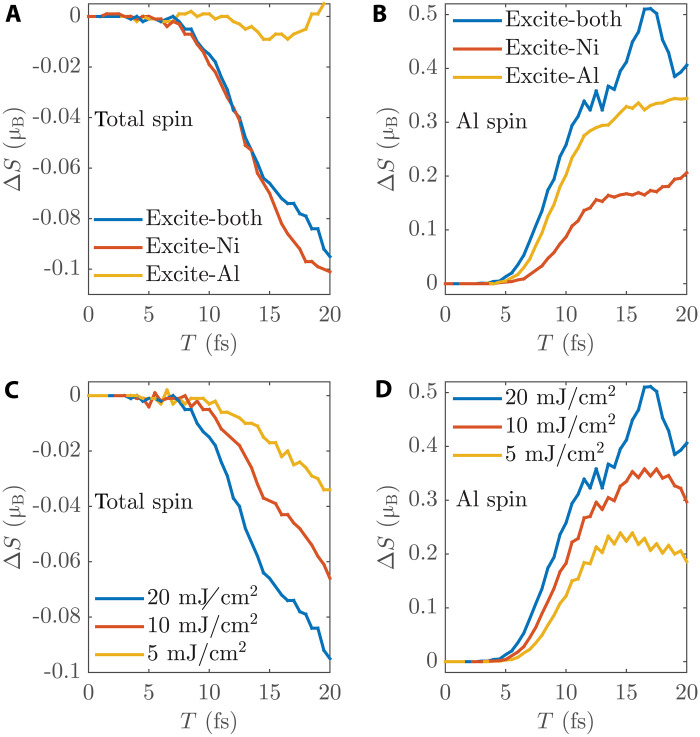
Spin evolution of the Ni_4_Al_12_ slab under different excitations. (**A** and **B**) Different excitation regions. (**C** and **D**) Different laser fluences. The *y*-axis value in (A) and (C) is the total spin change of the whole slab, while that in (B) and (D) is the Al spin change.

Figure 5 (A and B) compares their results for the total spin change and the Al spin change, respectively. As seen in Fig. 5A, the total spin evolution is similar to the original simulation when only Ni is excited, while there is almost no demagnetization when only Al is excited. This is because the total spin quenching of the whole slab is contributed only by SOC-induced spin dissipation, mainly through the SOC channel of Ni, which in turn is controlled by Ni excitation.

As for Al spin, its change comes from spin transport. Assuming that spin transport was dominated by the OISTR mechanism, there would be similar transport as in the original simulation when only Ni is excited, and there would be no spin transport when only Al is excited. However, our observations (Fig. 5B) are completely opposite. It can be seen that spin transport in the case of Al excitation is slightly smaller than in the original simulation, while that in the case of Ni excitation is remarkably reduced. Both are not zero. These observations fit well with the *sp*-*d* spin exchange picture. When only Ni is excited, there are much fewer free carriers and empty states for spin flipping in Al, which results in the reduced spin transport from Ni to Al. In contrast, when only Al is excited, the number of excited carriers in Al is retained and their spins can flip. But the Ni excitation is restricted in this situation, which limits the source side for spin exchange and leads to smaller spin transport. These results indicate that both Al and Ni excitation play a role, and Al excitation is even more important. We have detailed these comparisons in table S1. These distinctions between different excitations demonstrate the *sp*-*d* spin exchange physics mentioned above and also match well with many experiments where the laser is shone on different regions of FM/NM samples. We will discuss these experiments in detail later.

Because of the important role of carrier excitation, we can change the laser power to manipulate the spin dynamics. Figure 5 (C and D) illustrates the spin evolution under different laser fluences. As seen in Fig. 5C, the total spin dissipation decreases under lower excitation, indicating that SOC-induced dissipation is proportional to the laser fluence. Figure 5D shows that the spin transport is also proportional to the laser fluence. Overall, there is still a relatively large spin transport (e.g., ∼0.01 μ_B_/fs) at a smaller fluence (e.g., 5 mJ/cm^2^).

## DISCUSSION

We now turn to discuss the generality of the *sp*-*d* spin transport theory by studying the dependence on FM film thickness, laser parameters, and FM/NM atomic types, as well as connecting this theory to a variety of recent experiments.

**Fig. 6.**
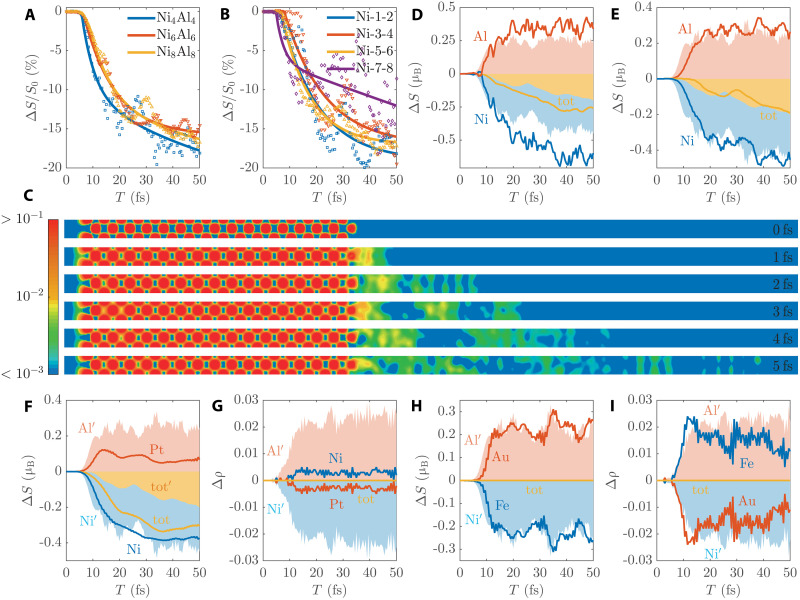
Dynamics in different cases. (**A**) Demagnetization rates of the whole Ni layers in three different systems. (**B**) Demagnetization rates of different Ni layers in Ni_8_Al_8_, where Ni-1-2 are the two surface Ni layers and Ni-7-8 are the two interface Ni layers. (**C**) Snapshots of the real-space spin density of Ni_32_Al_32_ (∼10 nm) excited by the laser with 2-fs duration. (**D** and **E**) Spin evolution of different regions of Ni_4_Al_4_ under the light of (D) 800-nm wavelength and (E) 30-fs duration, compared with the original simulation (shaded areas). Other laser parameters in the two simulations are kept as before. (**F**) Spin and (**G**) charge evolution of different regions of Ni_4_Pt_4_ (solid lines), compared with Ni_4_Al_4_ (shared areas, labeled with the Ni′, Al′, and tot′ symbols). (**H**) Spin and (**I**) charge evolution of different regions of Fe_4_Au_4_ (solid lines), compared with Ni_4_Al_4_ (shaded areas, labeled with the Ni′ and Al′ symbols), where SOC is turned off in both simulations.

First, to study the dependence on FM film thickness, we have performed two additional simulations by gradually increasing the thickness of Ni layers. One is a six-monolayer Ni film on a six-monolayer Al film (denoted as Ni_6_Al_6_), and the other is an eight-monolayer Ni film on an eight-monolayer Al film (denoted as Ni_8_Al_8_). We compare their results with the original Ni_4_Al_4_ system (under the same laser excitation as before), as shown in Fig. 6 (A and B) and fig. S15. It can be seen from Fig. 6A that the average Ni demagnetization rates for the three systems are very close, without obvious decay with the increase of Ni thickness. We can thus expect a similar demagnetization rate in even thicker films. Furthermore, like Ni_4_Al_12_ (Fig. 2B), all Ni layers in Ni_8_Al_8_ (Fig. 6B), Ni_6_Al_6_, and Ni_4_Al_4_ (fig. S15) have large demagnetization rates, despite some variations between different layers. The two surface layers in Ni_8_Al_8_ are even more demagnetized than the interface layers. This supports that the eventual *sp*-*d* exchange-induced spin transport can reach a long distance. These results give a sense of what the spin dynamics will be like in thicker films.

To get a more intuitive picture of long-range spin transport in thick films, we have simulated a very long slab: Ni_32_Al_32_ (∼10 nm). Since the computational cost of TDDFT simulations scales in *O*(*N*^3^), where *N* is the number of electrons, this calculation is extremely time consuming. Therefore, we use a short laser of 2-fs duration and perform the simulation for only 5 fs. We also reduce a little bit the accuracy (e.g., the energy cutoff) to save the computational cost. Figure 6C shows the evolution of real-space spin density of the whole slab, which demonstrates the long-range spin transport. Note that the film thicknesses of some representative experiments are also on the length scale we simulate. For example, the thickness of Ni layer in the Ni/Pt experiment (*3*) is 2 to 4 nm. The thickness of Co-Pt layer in the [Co-Pt]/Ru/[Co-Pt] experiment (*11*) is 4.4 nm.

Second, we study the dependence on laser parameters. We add two simulations by changing the laser wavelength and pulse width, respectively. The first one is changing the wavelength from the original 600 nm to 800 nm while keeping all other parameters (e.g., laser fluence) the same. The second one is changing the laser duration from the original 20 fs to 30 fs while keeping the other settings the same. Their results for the Ni_4_Al_4_ system are shown in Fig. 6 (D and E) and fig. S16. It is seen that there is a large spin flow from Ni to Al (Fig. 6, D and E), while the counterpart charge flow is much smaller (fig. S16). Figure S16 also illustrates the spin exchange process between Ni and Al. These results indicate that *sp*-*d* exchange-induced spin transport is a general light-spin phenomenon, not limited to a specific laser excitation.

Third, we study the dependence on the NM type of the substrate. As mentioned earlier, the SOC of Al is negligible. We are interested in how the spin dynamics would be on an NM with strong SOC. Here, we first examine the Ni/Pt system, which has been studied by a few ultrafast pump-probe experiments (*3*). We consider a four-monolayer Pt film on a four-monolayer Ni film (denoted as Ni_4_Pt_4_). The applied laser remains the same as in the simulations for Ni/Al in Figs. 2 and 3. Figure 6F shows the spin evolution of Ni_4_Pt_4_, in comparison with Ni_4_Al_4_. We see that the Ni spin change in Ni_4_Pt_4_ is slightly smaller than that in Ni_4_Al_4_, but they are relatively close. The Ni spin change is contributed by both spin dissipation from its own SOC channel and spin transport to the NM substrate. Because we use the same laser in Ni_4_Pt_4_ and Ni_4_Al_4_, the amplitude of spin dissipation in Ni should be close in both systems. Thus, we can deduce that the amplitude of spin transport in Ni_4_Pt_4_ is slightly smaller than in Ni_4_Al_4_, but should be relatively close. However, as can be seen from the spin curves of Pt and Al, the spin injection into Pt is only half of that into Al. It reaches the maximum at about 15 fs and then starts to fall with fluctuations. This sharp difference between Pt and Al is due to the strong SOC of Pt. Almost immediately after receiving the spin injection from Ni, the Pt spin has started to demagnetize through its SOC channel. Although Pt receives almost the same amount of spin injection as Al, it quickly dissipates into other angular momentum reservoirs (e.g., electron orbital). This strong spin dissipation in Pt is also confirmed in the curves of the total spin, where its change in Ni_4_Pt_4_ is much larger than that in Ni_4_Al_4_. Figure 6G further shows the charge evolution of Ni_4_Pt_4_, which indicates that the large spin transport from Ni to Pt is also a pure spin flow.

We also examine a completely different FM/NM system, i.e., a multilayer Fe/Au film, which has been investigated by many experiments (*14*, *67*, *79*–*81*). Similarly, we simulate a four-monolayer Fe film on a four-monolayer Au film (denoted as Fe_4_Au_4_). We expect that it will be very similar to Ni_4_Pt_4_, i.e., spin injection from Fe and spin dissipation by SOC will compete inside the Au layer. To show how spin exchanges between Fe and Au, here we perform a different simulation with SOC turned off. This will disable SOC-induced spin dissipation in both Fe and Au. In addition, because the light absorption coefficient of Au is about 2.5 times smaller than that of Al at 600 nm (*82*, *83*), we increase the incident laser fluence by 2.5 times while keeping all other settings the same. This will help us compare Fe_4_Au_4_ and Ni_4_Al_4_. Figure 6 (H and I) show the results. It is seen that there is a precise spin-exchange process between Fe and Au with their total spin conserved. The amplitude of spin transport is close to that in Ni_4_Al_4_. The corresponding charge flow is also very tiny, but in the opposite direction from Au to Fe. These results indicate a pure spin flow from Fe to Au.

With all of these results (Figs. 2 to 6), we now can connect our simulations to many existing experiments. In the following, we choose several classic experiments from the literature as examples, which cover the works with different FM/NM systems and different pumping/probing settings (e.g., excitations on different regions) as well as the comparisons between different types of systems (e.g., FM/NM films, FM/insulator films, and FM bulk). They represent important milestones in the field of light-induced ultrafast spin dynamics.

1) In (*32*), Eschenlohr *et al.* investigated two samples. The first is a 20-nm Ni layer on the Al foil, and the second is a 30-nm Au layer on top of a 15-nm Ni layer. Strong Ni demagnetization was observed in both samples under laser excitation. The first sample is what we have discussed in Figs. 2 to 5. Its Ni demagnetization is contributed by both SOC-induced spin dissipation and strong spin transport to the Al substrate. As for the second sample, most of the incident light is absorbed by the Au and cannot penetrate into the Ni. Approximately, we can assume that only the nonmagnetic Au region is excited, which is similar to only exciting the nonmagnetic Al region in the Ni/Al system as studied in Fig. 5 (A and B) (yellow curves). Therefore, the Ni demagnetization in this sample can be explained by the same *sp*-*d* spin transport theory as seen in Fig. 5 (A and B). That is, Au excitation generates a large number of free carriers to involve in the *sp*-*d* spin exchange with Ni, which results in spin transport from Ni to Au. Such spin flow leads to the observed large Ni demagnetization.

2) Similar phenomena have been found in (*59*), where Bergeard *et al.* investigated a series of samples with a thick Au capping layer on top of a thin Co-Pt layer. Most light cannot penetrate into Co-Pt, but it still undergoes rapid and strong demagnetization. This observation is also consistent with our simulations in Fig. 5 (A and B) (yellow curves). This type of experiment cannot be explained by the OISTR mechanism (Fig. 1B), which requires the optical excitation of FM. As demonstrated in Fig. 5 (A and B), our theory does not have such a limitation, and *sp*-*d* exchange-induced spin transport can be triggered by exciting FM, NM, or both of them. Even with only NM optically excited, there is still a large spin flow from FM to NM. This covers a wide range of experimental works that are hardly explained by previous ab initio theories.

3) In (*3*), Siegrist *et al.* compared light-induced spin dynamics in two different samples: (A) Ni bulk and (B) Ni/Pt multilayer thin film. The pump-probe experiments found that the Ni/Pt sample has a larger Ni demagnetization than the bulk Ni. This result is what we have simulated in Fig. 6 (F and G) (also similar to Fig. 2A). The spin exchange between Ni and Pt causes a large and pure spin flow from Ni to Pt, which provides an additional channel for Ni demagnetization and results in the larger observation. Our *sp*-*d* spin exchange theory can well explain this type of experiment for the comparison between FM bulk systems and FM/NM multilayer thin-film systems.

4) In (*14*), Melnikov *et al.* fabricated several epitaxial Au/Fe/MgO(001) samples [e.g., Au(50 nm)/Fe(15 nm)/MgO(001)]. The pumping light is back shone into the Fe from the transparent MgO substrate, and the probing light is examined at the front Au surface. They observed spin polarization and a magnetic hysteresis loop at the Au surface from around 30 fs. In (*79*), Kampfrath *et al.* also demonstrated a light-induced spin current from Fe to Au in a Fe(10 nm)/Au(2 nm) sample, which was measured by the inverse spin Hall effect and terahertz emission. Both experiments match with our simulations in Fig. 6 (H and I). Strong *sp*-*d* spin exchange coupling leads to the pure spin transport from Fe to Au.

5) In (*67*), Razdolski *et al.* compared three different samples: (A) an 8-nm-thin Fe layer on a 130-nm Au layer, (B) a 34-nm-thick Fe layer on an 130-nm Au layer, and (C) an 8-nm-thin Fe layer on the insulating MgO substrate. The pump-probe experiments showed that the demagnetization rate of sample A is higher than that of sample B, and both are higher than that of sample C. This result is consistent with the *sp*-*d* spin transport theory. In samples A and B, *sp*-*d* spin exchange interaction exists between Fe and Au, and can lead to spin transport from Fe to Au. In contrast, spin transport is restricted in the insulating MgO substrate of sample C because the lack of free carriers in MgO prevents the *sp*-*d* spin exchange. Consequently, the Fe demagnetization of samples A and B is contributed by both spin dissipation and spin transport, while sample C is contributed only by spin dissipation. This can explain why samples A and B exhibit a larger demagnetization than sample C. As for the comparison between samples A and B, it supports our simulations of Fig. 5 (A and B) (red and blue curves). In sample B with 34-nm Fe, most of the incident light cannot reach Au because the light penetration depth of Fe is about 40 nm (*84*). This is similar to exciting only the Ni region in the Ni/Al system (red curves of Fig. 5, A and B). As a result, there are fewer free carriers in Au involved in the *sp*-*d* spin exchange, leading to a smaller amount of spin flow from Fe to Au. In contrast, in sample A with 8-nm Fe, more light can enter Au and can excite more free carriers in Au, leading to a larger amount of *sp*-*d* spin exchange between Fe and Au. This explains why the demagnetization in sample A is greater than that in sample B.

In addition, for the same 8-nm Fe in samples A and C, both the relative rotation variation and the ellipticity variation of sample A are about twice the values of sample C. This implies that the spin transport channel contributes half of the demagnetization in sample A. We also notice that sample A exhibits a very rapid spin decay that mostly occurs within 50 fs, whereas sample C exhibits a relatively slower one that lasts over 200 fs. This indicates that the major spin transport in sample A happens on a very short timescale, consistent with our simulations (Figs. 2C and 3). These results give a sense of the contribution and timescale of spin transport, and also provide good illustrations for the comparisons between FM/NM systems and FM/insulator systems.

In addition to the above experiments, the *sp*-*d* exchange-induced spin transport theory can be used in the interpretation of many more ultrafast spin dynamics phenomena in multiplayer metallic thin films. We have provided several more instances in the Supplementary Materials. Note that some of these experiments have been previously explained by other theories. Our *sp*-*d* spin exchange theory provides a different and unified picture.

In conclusion, we have unveiled the underlying physics for light-induced ultrafast spin transport in multiplayer metallic films. We found that the *sp*-*d* spin exchange interaction can induce an ultrafast, large, pure, and long-range spin current from FM to NM, resulting in spin dynamics consistent with most experiments on the femtosecond timescale. It is able to explain a variety of ultrafast light-spin manipulation experiments, providing a unified picture for them. We anticipate that it will open a way for faster manipulation of magnetic states (e.g., by controlling the coupling between different FM and NM as well as by controlling the carrier excitations).

## METHODS

Our RT-TDDFT adopts the following noncollinear time-dependent Kohn-Sham equation to describe the spin, electron, and structure dynamics:

$$i\frac{\partial }{\partial t}\left[\begin{array}{c} \psi_{ik}^{↑}(r,t)\\ \psi_{ik}^{↓}(r,t) \end{array}\right]=H(r,t)\left[\begin{array}{c} \psi_{ik}^{↑}(r,t)\\ \psi_{ik}^{↓}(r,t) \end{array}\right]$$ (1)

$$H(r,t)=\frac{1}{2}{\left[-i\nabla +A(r,t)\right]}^{2}+V(r,t)+\frac{1}{2}\sigma \cdot B(r,t)$$ (2)

$$V(r,t)=V_{\mathrm{ion}}(r,t)+V_{H}(r,t)+V_{\mathrm{XC}}(r,t)+V_{\mathrm{SOC}}(r,t)+V_{\mathrm{ext}}(r,t)$$ (3)

where the noncollinear wave function contains spin-up $\psi_{ik}^{↑}(r,t)$ and spin-down $\psi_{ik}^{↓}(r,t)$ components, *i* is the band index, and *k* is the *k*-point index. *A*(*r*, *t*) and *B*(*r*, *t*) represent the magnetic vector potential and magnetic field of the incident laser; σ is the Pauli matrix vector [σ*_x_*, σ*_y_*, σ*_z_*]. The Hamiltonian *H*(*r*, *t*) in Eqs. 1 and 2 is a 2 × 2 matrix, consisting of the kinetic energy term, laser potential term (i.e., *A* and *B* fields), and *V*(*r*, *t*) term. *V*(*r*, *t*) includes the ionic potential *V*_ion_, Hartree potential *V*_H_, exchange-correlation potential *V*_XC_, SOC potential *V*_SOC_, and other external potential *V*_ext_. The charge density ρ(*r*, *t*) is also a 2 × 2 matrix with the formula as below:

$$\rho (r,t)=\left[\begin{array}{cc} \rho_{\alpha \alpha }(r,t) & \rho_{\alpha \beta }(r,t)\\ \rho_{\beta \alpha }(r,t) & \rho_{\beta \beta }(r,t) \end{array}\right]$$ (4)

$$\rho_{\alpha \beta }(r)=\sum_{ik}o(i,k)\cdot \psi_{ik}^{\alpha }(r,t)\cdot \psi_{ik}^{\beta }(r,t)$$ (5)

where α and β indicate spin-up or spin-down components, and *o*(*i*, *k*) is the occupation number of the state ψ*_ik_*(*r*, *t*). Different interactions can then be written as a function of ρ(*r*, *t*). For example, the Hartree term and exchange-correlation term are represented as:

$$V_{H}(r,t)=\int \frac{\rho_{\alpha \alpha }(r^{\prime },t)+\rho_{\beta \beta }(r^{\prime },t)}{|r-r^{\prime }|}d^{3}r^{\prime }$$ (6)

$$\begin{array}{cc} V_{\mathrm{XC}}(r,t)= & \frac{\delta E_{\mathrm{XC}}}{\delta [\rho_{\alpha \alpha }(r,t)+\rho_{\beta \beta }(r,t)]}+\\ & \frac{1}{2}\left[\frac{\delta E_{\mathrm{XC}}}{\delta \rho_{1}(r,t)}-\frac{\delta E_{\mathrm{XC}}}{\delta \rho_{2}(r,t)}\right]\cdot m(r,t)\cdot \sigma \end{array}$$ (7)

where ρ_1_(*r*, *t*) and ρ_2_(*r*, *t*) are the spin-up and spin-down magnitudes of the charge density matrix ρ(*r*, *t*) at its principle direction *m*(*r*, *t*) at the point *r*. *E*_XC_ is the exchange-correlation energy. Note that *V*_XC_ is treated at the functional level [like many existing RT-TDDFT codes (*63*)] and does not include the memory effect (*31*).

The incident laser in our method is treated as an external electric-magnetic field [i.e., *A*(*r*, *t*) and *B*(*r*, *t*)] in the Hamiltonian. Because the laser wavelength (several hundred nanometers for the visible light) is much longer than the simulated supercell size (several nanometers), we apply the dipole approximation and neglect the spatial dependence of the laser field *A*(*r*, *t*) and *B*(*r*, *t*). The temporal dependence of the laser is represented by a sinusoid multiplied by a Gaussian envelope (see the Supplementary Materials for details).

To implement the time evolution for Eqs. 1 to 3, the conventional approach is to expand the wave functions on plane-wave basis functions (or atomic basis functions) and perform the direct integration with a very small time step. This will cause a huge number of integration steps, and each step requires several large-scale matrix computations (e.g., *H*ψ*_ik_* and fast Fourier transform). Such heavy computational cost limits the application of these conventional approaches in spin dynamics simulations.

To speed up the time evolution, our RT-TDDFT algorithm uses dynamical adiabatic states to expand the wave functions and uses the iterative leap-frog method to perform the integration. The combination of both techniques can increase the integration time step from a typical 10^−5^ fs to 0.05 fs and can avoid time-consuming large-scale matrix computations at each step, thus achieving a large acceleration over previous RT-TDDFT methods.

Specifically, first, the time-dependent wave function ψ*_ik_*(*r*, *t*) during the time step from *t*_1_ to *t*_2_ = *t*_1_ + Δ*t* is expanded by the adiabatic states ϕ*_lk_*(*r*, *t*_1_) at *t*_1_:

$$\left[\begin{array}{c} \psi_{ik}^{↑}(r,t)\\ \psi_{ik}^{↓}(r,t) \end{array}\right]=\sum_{l}C_{il}^{k}(t)\cdot \left[\begin{array}{c} \phi_{lk}^{↑}(r,t_{1})\\ \phi_{lk}^{↓}(r,t_{1}) \end{array}\right]$$ (8)

$$H[\rho (r,t_{1})]\left[\begin{array}{c} \phi_{lk}^{↑}(r,t_{1})\\ \phi_{lk}^{↓}(r,t_{1}) \end{array}\right]=\varepsilon_{lk}(r,t_{1})\left[\begin{array}{c} \phi_{lk}^{↑}(r,t_{1})\\ \phi_{lk}^{↓}(r,t_{1}) \end{array}\right]$$ (9)

where ρ(*r*, *t*_1_) is the charge matrix at *t*_1_ and *H*[ρ(*r*, *t*_1_)] is the corresponding Hamiltonian matrix. Then, the time-dependent Kohn-Sham equations (Eqs. 1 to 3) can be rewritten as:

$$\partial C_{il}^{k}(t)/\partial t=-i\sum_{j}C_{ij}^{k}(t)H_{lj}^{k}(t)$$ (10)

$$H_{lj}^{k}(t)=\langle \phi_{lk}(r,t_{1})|H[\rho (r,t)]|\phi_{jk}(r,t_{1})\rangle$$ (11)

The dimension of the coefficient $C_{ij}^{k}$ matrix and Hamiltonian $H_{lj}^{k}$ matrix depends on the number of electrons of the studied systems, and the value is usually several hundred for most cases. It is about 1% or less of the counterpart value when we expand directly ψ*_ik_* on a plane-wave basis set. This will remarkably reduce the cost of matrix multiplication and diagonalization during the integration. It is one major advantage of the adiabatic state basis.

Second, in the integration, we have to compute $H_{lj}^{k}(t)$ (Eqs. 10 and 11). It is very time consuming to directly compute ρ(*r*, *t*), *H*[ρ(*r*, *t*)], and then $H_{lj}^{k}(t)$ step by step for every *t* (*t* = *t*_1_ + *i* · *dt*; *dt* = Δ*t*/*n* is the sub-time step) along *t*_1_ to *t*_2_. Instead, we use leap-frog prediction and linear interpolation to compute $H_{lj}^{k}(t)$, as below:

$$H_{lj}^{k}(t)=[1-a(t)]\cdot H_{lj}^{k}(t_{1})+a(t)\cdot H_{lj}^{k}(t_{2})$$ (12)

$$a(t)=(t-t_{1})/(t_{2}-t_{1})$$ (13)

$H_{lj}^{k}(t_{2})$ is obtained by an iterative leap-frog prediction of ρ(*r*, *t*_2_). To be specific, we first use extrapolation to predict the initial ρ(*r*, *t*_2_) [denoted as ρ_0_(*r*, *t*_2_)] by the previous ρ(*r*, *t*) trajectory, and compute $H_{lj}^{k}(t_{2})$. Then we perform the integration from *t*_1_ to *t*_2_ by Eqs. 10 to 13 and obtain the coefficient $C_{il}^{k}(t_{2})$, by which we have ψ*_ik_*(*r*, *t*_2_) and the new ρ(*r*, *t*_2_) [denoted as ρ′_1_(*r*, *t*_2_)]. We take the difference between the two ρ(*r*, *t*_2_) as the convergence criterion. If it is large, we use charge matrix mixing (including extended Kerk mixing and Pulay mixing) to predict a new ρ(*r*, *t*_2_) [denoted as ρ_1_(*r*, *t*_2_)], and repeat the integration procedure. The extended Kerk mixing and Pulay mixing are similar to those in self-consistent electronic structure calculations. We find that they are better than the simple linear mixing, e.g., ρ_1_(*r*, *t*_2_) = (1 − β)ρ_0_(*r*, *t*_2_) + βρ′_1_(*r*, *t*_2_), where β ∈ (0,1) is the mixing parameter. We repeat the integration, charge mixing, and $H_{lj}^{k}(t_{2})$ calculation several times until the ρ(*r*, *t*_2_) is converged. This leap-frog way makes the calculation of $H_{lj}^{k}(t)$ easy. Together with the advantage of the small dimension of $H_{lj}^{k}(t)$ and $C_{ij}^{k}$ matrices, the computational cost for the integration from *t*_1_ to *t*_2_ is negligible compared to the calculation of $H_{lj}^{k}(t_{2})$, even if we use a very small sub-time step (e.g., *dt* = Δ*t*/1000). After the convergence, we then compute the Hellmann-Feynman atomic forces and move the ions in Ehrenfest dynamics using the classical Verlet algorithm, as in the conventional molecular dynamics simulation. This will include the average effect of various phonon modes. This is one time step (from *t*_1_ to *t*_2_) for the evolution of the whole system. We will move to the next time step (from *t*_2_ to *t*_3_) and use ${\left[\phi_{lk}^{↑}(r,t_{2})\;\phi_{lk}^{↓}(r,t_{2})\right]}^{T}$ as the new basis set to expand ${\left[\psi_{ik}^{↑}(r,t)\;\psi_{ik}^{↓}(r,t)\right]}^{T}$. The whole procedure is repeated until reaching the preset simulation time limit.

The above RT-TDDFT algorithm and the preliminary ground-state calculations were implemented in our noncollinear magnetic version of plane-wave density function theory calculation code (PEtot). The adiabatic state wave function was expanded by a plane-wave basis set, and norm-conserving pseudopotential was used. The 4 × 4 × 1 *k*-point mesh without symmetry was used for the summation over the Brillouin zone for long slab systems.

## Supplementary Materials

### This PDF file includes:

Sections S1 to S16 Figs. S1 to S16 Table S1 Legend for movie S1 References
