# Supplementary material for: Light-induced ultrafast spin transport in multilayer metallic films originates from sp-d spin exchange coupling
Source: Sci Adv. 2023 Dec 15;9(50):eadi1618. doi: 10.1126/sciadv.adi1618 (PMC10848703; doi:10.1126/sciadv.adi1618)
Supplement: Supplementary file 1 — Sections S1 to S16 Figs. S1 to S16 Table S1 Legend for movie S1 References [file sciadv.adi1618_sm.pdf]

Science Advances
AAAS

Supplementary Materials for

Light-induced ultrafast spin transport in multilayer metallic films originates from *sp*-*d* spin exchange coupling

Zhanghui Chen *et al.*

Corresponding author: Zhanghui Chen, zhanghuichen@semi.ac.cn; Lin-Wang Wang, lwwang@semi.ac.cn

Sci. Adv. **9**, eadi1618 (2023)
DOI: 10.1126/sciadv.adi1618

The PDF file includes:

Sections S1 to S16
Figs. S1 to S16
Table S1
Legend for movie S1
References

Other Supplementary Material for this manuscript includes the following:

Movie S1

1. Laser field

In our simulations, the dipole approximation is applied to the laser electric-magnetic field, and the spatial dependence of $A(r,t)$ and $B(r,t)$ is ignored. With this approximation, the high-order terms (e.g., electric quadrupole transition matrix) of the laser field are zero. If we treated $B(r,t)$ as $\nabla \times A(r,t)$, $B(r,t)$ would also become zero. Because both the electric field ($E(r,t) = \partial A(r,t)/\partial t$) and magnetic field ($B(r,t)$) of the femtosecond laser are very large in many experiments, we treat them separately (so the $B(r,t)$ field cannot be written as $\nabla \times A(r,t)$).

The temporal dependence of the laser field in our RT-TDDFT method is represented by a sinusoid multiplied by a Gaussian envelope, as below:

$$A\left(r,t\right) = A_0 \frac{e^{-(t-t_{\mathrm{p}})^2/2\sigma^2}}{\sqrt{2\pi}\sigma} \sin\left(w\left(t-t_{\mathrm{p}}\right)+\phi\right) \tag{1}$$

$$B\left(r,t\right) = B_0 \frac{e^{-(t-t_{\mathrm{p}})^2/2\sigma^2}}{\sqrt{2\pi}\sigma} \cos\left(w\left(t-t_{\mathrm{p}}\right)+\phi\right) \tag{2}$$

where the parameters A_0 and B_0 are the amplitudes; t_{p} is the peak time; σ is the full width at half maximum (FWHM) divided by $2\sqrt{2\ln 2}$; w is the frequency; and ϕ is the initial phase. The duration (t_{tot}) of the laser is defined as the period with non-zero A and B values. In the simulations for this paper, it is set to the time from 0 fs to t_{tot}. Outside of this period, we manually set the A and B values to zero. t_{p} is set to the center of this period.

In the main simulations for the Ni/Al systems, we used the laser of 600 nm wavelength. The wavelength of 600 nm corresponds to 2 fs in one cycle. The laser of 20-fs duration used in the main simulations has exactly 10 cycles. This is easy for the theoretical treatment of the laser pulse. The peak time t_{p} is 10 fs, and the conventional FWHM duration is 8 fs here. In the later simulations, we also tested the effects of different laser parameters (e.g., fluences, wavelengths, and durations). In all simulations, the laser propagation direction is along the initial Ni magnetic

moment direction, and the laser polarization is perpendicular to the magnetic moment direction.

Fig. S1 shows the temporal profile of the laser magnetic vector potential $A(r,t)$ used in the main RT-TDDFT simulations.

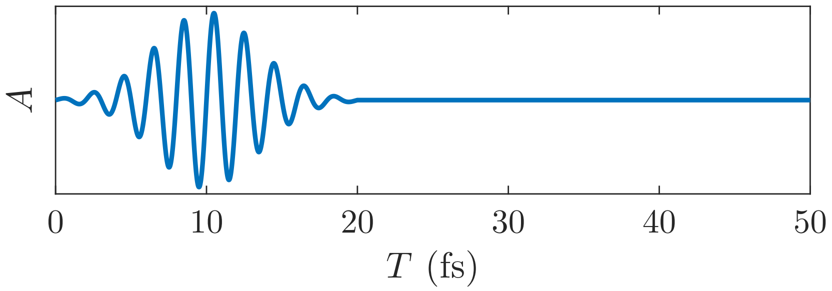

Fig. S1. The magnetic vector potential (A) of the applied laser field.

2. Comparisons between RT-TDDFT and Elliott-Yafet scattering methods

In our RT-TDDFT simulations, we have included all the important interactions (e.g., electron-electron and electron-phonon) in the Hamiltonian. Compared to many parameterized (or analytical) treatments, our Hamiltonian is ab initio. For a detailed comparison, we take the well-known Elliott-Yafet electron-electron scattering method (*64*) as an example. Its potential for two-electron scattering (from $|n_1,k_1\rangle$ to $|m_1,k_1+q\rangle$ and from $|n_2,k_2\rangle$ to $|m_2,k_2-q\rangle$) is usually described by the following approximation (*64*):

$$V^{n_1m_1}_{n_2m_2}(\vec{k}_1,\vec{k}_2,\vec{q},\omega)=f^{m_1}_{n_1}(\vec{k}_1,\vec{q})\cdot f^{m_2}_{n_2}(\vec{k}_2,\vec{q})\cdot v(q)\cdot\varepsilon^{-1}(q,\omega) \tag{3}$$

where

$$f^{m_1}_{n_1}(\vec{k}_1,\vec{q})=\langle n_1,\vec{k}_1|m_1,\vec{k}_1+\vec{q}\rangle \tag{4}$$

$$f^{m_2}_{n_2}(\vec{k}_2,\vec{q})=\langle n_2,\vec{k}_2|m_2,\vec{k}_2-\vec{q}\rangle \tag{5}$$

$$\hbar\omega=E(n_1,\vec{k}_1)-E(m_1,\vec{k}_1+\vec{q}) \tag{6}$$

Here n_1, m_1, n_2, and m_2 are band indexes; k_1 and k_2 are k-point vectors; q is the k-point vector change during the scattering; $E(n_1, k)$ and $E(m_1, \vec{k}_1 + \vec{q})$ are the eigen energies; $v(q)$ is the bare coulomb potential and $\varepsilon^{-1}(q, \omega)$ is the inverse dielectric function. In Elliott-Yafet electron-electron simulations, $f_{n_1}^{m_1}(\vec{k}_1, \vec{q})$ is usually approximated as 1 if $n_1 = m_1$ and as a (like a Yafet parameter) if $n1 \neq m1$ (*64*). $f_{n_2}^{m_2}(\vec{k}_2, \vec{q})$ is similar. Spherical symmetry is also used in the approximation of eigen energies:

$$E(n_1, \vec{k}_1) = E(n_1, |\vec{k}_1|) \tag{7}$$

Compared to this Elliott-Yafet electron-electron potential, the electron-electron interaction in our method (i.e., the Hartree V_{H} term and the exchange-correlation V_{XC} term in Eqs. (1-3) of the main text) is computed directly from the real-time wave functions and charge density (Eqs. (4-7) of the main text) without these approximations. We also update the electron-electron interaction with the time evolution of wave functions, rather than using the fixed values from the initial wave functions throughout the simulations. Such accurate calculations and real-time updating of the electron-electron interaction are advantages of RT-TDDFT methods and are not possible in previous analytical models.

In the Elliott-Yafet electron-electron interaction, its contribution to the dynamics and the demagnetization is described by the classical Boltzmann equation in the form like:

$$\frac{\partial occ(n_1, \vec{k}_1)}{\partial t} \propto \sum_{\vec{q}} \sum_{\vec{k}_2} \sum_{m_2} \sum_{n_2} \sum_{m_1} |V_{n_2 m_2}^{n_1 m_1}(\vec{k}_1, \vec{k}_2, \vec{q}, \omega)|^2 \tag{8}$$

Our RT-TDDFT method does not employ such a way. Instead, we compute the electron-electron interactions and the full Hamiltonian from the current wave functions and charge density. After that, we use the non-collinear time-dependent Kohn-Sham equation (Eqs. (1-3) of the main text) to update the wave functions, which are then used to calculate all the properties (e.g., spin and charge). This process is repeated to evolve the dynamics (see the Methods section in the main text).

This RT-TDDFT approach is very different from previous models (including the Elliott-Yafet electron-electron and Elliott-Yafet electron-phonon models). Different types of interactions (e.g., electron-electron, electron-phonon, spin-orbit, light-spin, and light-orbital) are fully included in the dynamics without preset assumptions. Electron-electron and electron-phonon scatterings are not parameterized as in previous methods. One major disadvantage of RT-TDDFT simulations is that they are too time-consuming, which is one reason why previous RT-TDDFT approaches are rarely used in spin dynamics simulations of complicated systems. Our new algorithm remarkably reduces the computational cost and makes spin dynamics simulations of relatively large systems affordable.

3. FM/NM films

Our studies have considered an abrupt contact to model the interface between FM and NM. Fig. S2 shows the Ni_4Al_{12} heterojunction structure used in our RT-TDDFT simulations. It consists of four monolayers of Ni packed on top of twelve monolayers of Al. Ni atoms are placed on the hollow site of the Al(001) surface, which is the most stable among all the possible contact sites (*85–87*). The structures of Ni_4Al_4, Ni_4Al_6, and Ni_4Al_8 are similar to Ni_4Al_{12}, while the isolated Ni_4 slab has the same configuration as the Ni in these heterojunctions. All these slab structures are relaxed to their stable minima by the conjugate gradient algorithm.

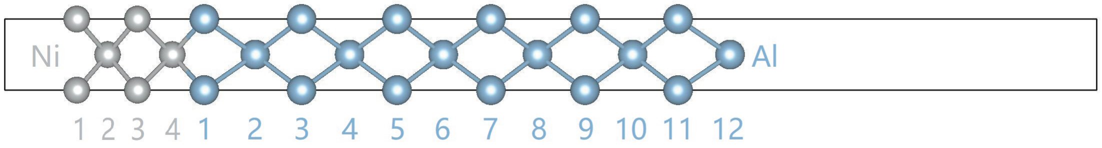

Fig. S2. The Ni_4Al_{12} heterojunction structure. It consists of 4 monolayers of Ni and 12 monolayers of Al plus a vacuum layer of >15 Å.

It should be noted that the lattice parameters of Ni and Al are not well matched. Previous studies have shown that some intermetallic compounds (e.g., Ni_3Al) exist in the interface region

of Ni/Al(001) films (*85, 87*). In order to describe such a complicated interface feature, a very large supercell (e.g., ∼7,000 (*87*), ∼16,000 (*88*), or ∼200,000 (*89*) atoms) is needed. The dynamics of such a large system is usually described by classical embedded-atom potentials (*87–90*), and is neither affordable in the common ab initio simulations nor in the RT-TDDFT simulations. Because the intermetallic compounds still maintain metallic contact and strong *sp–d* exchange coupling, it should not change the dominant spin transport mechanism in this work. Thus, it is reasonable to use an abrupt contact with Ni-Al bonds to simplify the interface model.

4. Material parameters

In our RT-TDDFT simulations, there are several types of material parameters. These parameters should be called choices for numerical methods and approximations in ab initio simulations. The first is the pseudopotential for each atomic type. There are three common choices: norm-conserving, ultrasoft, and projector-augmented-wave. All of them have been well built up for the common elements in the element tables. We have chosen norm-conserving pseudopotential here. The second is the exchange-correlation functional. We have chosen the noncollinear version of local spin density approximation. The third are the atomic structures and the slab supercell parameters. The initial geometry is taken from the experiments. We then relax the atomic positions and the supercell using ab initio calculations.

Our RT-TDDFT simulations do not need other material parameters, e.g., spin-resolved density of states, spin lifetime, and conductivity, which are required in many classical or semiclassical models (*49, 68, 69*). Many of these parameters can be obtained from ab initio simulations. For example, Figs. 3H and 3I show the calculated spin-resolved density of states. This is an advantage of RT-TDDFT over other models. We do not need to adjust these parameters to match the experimental results. The results match the experiments naturally, which can verify

the correctness of the simulation.

5. Oscillated data points and data fitting procedure

In Figs. 2, 6A and 6B of the main text, the oscillated data points output by RT-TDDFT simulations are fitted with smooth curves to show the demagnetization trend. Considering that there are two terms (spin dissipation and spin transport) contributing to the Ni demagnetization, we employ the combination of two exponential decay functions to represent these curves, as below:

$$f(t) = a_1 \cdot \exp[-b_1 \cdot (t - c_1)] + a_2 \cdot \exp[-b_2 \cdot (t - c_2) + d] \tag{9}$$

where a_1, b_1, c_1, a_2, b_2, c_2 and d are fitting parameters. In addition, it can be seen from Figs. 2, 6A and 6B that there is a time delay (about 5 fs) between spin decay and laser excitation. Thus, only the data points after spin decay are imported into the fitting procedure. We employ the mean square error as the fitting criterion:

$$E = \sum_t [f(t) - S(t)]^2 / N_t \tag{10}$$

where $S(t)$ is the spin produced by RT-TDDFT simulations; $f(t)$ is the fitting value; and N_t is the number of data points. This fitting procedure is also applied to the results in this supplementary document.

There are two types of reasons for this oscillation. The first is the numerical computation reason. We used a relatively abrupt boundary (rather than a slowly varying smooth boundary) to estimate the contribution in each region. This can exactly compute the spin in each region, but it will also cause frequent but relatively small fluctuations during the dynamics. In addition, the oscillation might also be related to the k-point mesh. In our simulations, we use a $4\times4\times1$ k-point mesh without symmetry (i.e., 16 k-points) for the slab TDDFT simulations. Here, we add a test with an $8\times8\times1$ k-point mesh without symmetry (i.e., 64 k-points). The symmetry is

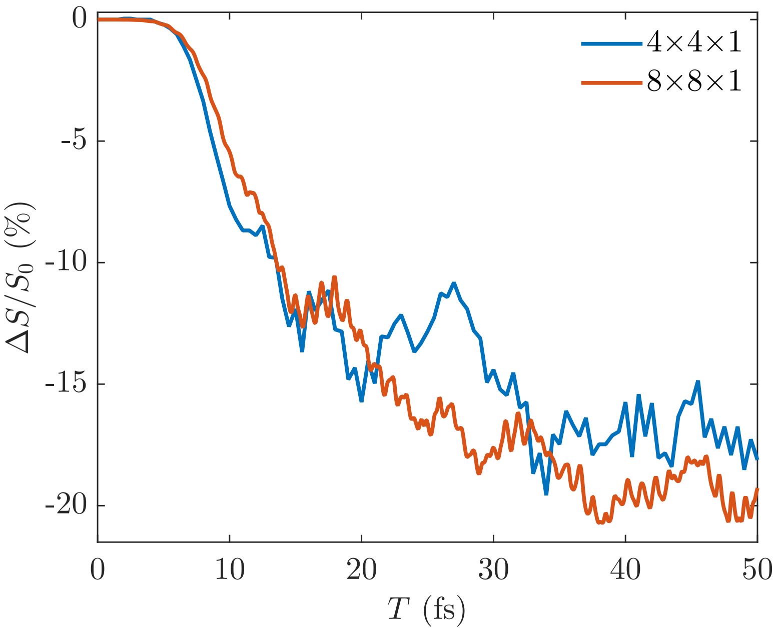

Fig. S3. The evolution of the Ni demagnetization rate in the two cases with different k-point meshes.

broken by the laser field and atomic motions. Their Ni spin dynamics for the Ni_4Al_4 system are compared in Fig. S3. It can be seen that the trends of the two curves are overall similar. This indicates that our simulations are reliable. The oscillation is still present in the curve with the 8×8×1 k-point mesh. Nevertheless, the oscillation amplitude is reduced in the dense k-points. This seems to indicate that the exact spin oscillation depends on the k-points, and thus might be partially due to an artifact. We also suspect that a larger supercell with more atoms might also reduce this oscillation. Unfortunately, due to the expensiveness of these calculations, it is difficult to perform the TDDFT simulations for very dense k-points and very large systems.

Note that in previous TDDFT simulations for very thin films by other groups (e.g., Ref. 3), the spin dynamics curves also show frequent oscillations.

Second, we suspect that there are more fundamental physical reasons. Frankly, we feel much more careful study and statistical averaging are needed to carefully identify the physical

origin. Nevertheless, we provide some speculations here. Note that these time oscillations exist only in FM/NM heterostructures, and do not exist in the common RT-TDDFT simulations for bulk systems. We thus suspect they might result from some interference and scattering effect due to the interface and surface. Since it is the total spin in each system (instead of a single wave function), it might have a mechanism similar to the Friedel oscillation from the metal surface or interface. Some recent literature has reported Friedel oscillation and dynamical Friedel oscillation in magnetic surfaces and interfaces. For example, Mitsui et al. (*91*) have observed the layer-by-layer magnetization oscillation with the depth below the surface of Fe(001)/MgO, and *s*–*d* exchange interaction plays an important role. Stephanovich et al. (*92*) have demonstrated the temporally oscillated spin dynamics in the topological insulator surface due to RKKY interactions (mediated by *sp*–*d* interaction). Stephanovich et al. (*93*) have also illustrated ultrafast Friedel oscillated spin current and associated RKKY interaction in the metal and semiconductor surface or interface. Our results are to some extent like these findings and are also driven by *sp*–*d* exchange interaction. But as mentioned above, at this stage, these are just some speculations.

In order to illustrate the fluctuated spin flow more clearly, we have combined the spin density figures into a video with a time resolution of 2 frames per fs (an output setting in our original simulations), as provided in the Supporting Video. It is seen that there is a fluctuated forward spin flow to the surface and an opposite reflective spin flow from the surface. Both flows mix together and cause a large spatial and temporal oscillation. Because there is no such oscillation in the demagnetization of bulk systems, we think the oscillation has no dependence on SOC-induced spin relaxation but is mainly due to the *sp*–*d* spin exchange between FM and NM.

We would like to note that there are a few recent experimental works for FM/NM thin films that show strongly oscillated spins. For example, Ref. 3 studied the laser-induced Ni spin dynamics in the Ni/Pt sample with a probing time resolution of less than 0.3 fs. It was

found that the Ni spin decreases with large oscillations. Nevertheless, it is unknown whether these oscillations are due to measurement noises or some fundamental physical reasons. Such oscillated spins are rarely discussed in earlier experiments. There could be several possible reasons. First, the time resolution of measurements is over 10 fs in many experiments, which might not capture these oscillations. Second, most films are relatively thicker, and the surface effect and interface effect are not as remarkable as in very thin films as in Ref. 3. While we deduce that these oscillations are related to the surface effect and interface effect, as we mentioned above.

6. Notes on the mismatch problem between theory and experiment

The demagnetization rate of the isolated Ni_4 film is about 3%, which is much smaller than the experimental observations (>30%). There are two reasons.

First, this demagnetization amplitude is sensitive to the laser wavelength in this very thin slab due to the quantum confinement effect. In the original simulation, we used a laser of 600 nm wavelength. The wavelength of 600 nm corresponds to 2 fs in one cycle. The laser of 20-fs duration used in the main simulations has exactly 10 cycles. This is easy for the theoretical treatment of the laser pulse. Here, we add a simulation with 800 nm wavelength. The results are shown in Fig. S4. We can see that the demagnetization rate of the Ni_4 film is much more remarkable, reaching about 9%, larger than the 3% in the case of 600 nm wavelength. The Ni demagnetization rate of the Ni_4Al_4 slab is about 26%, also larger than the 17% in the case of 600 nm wavelength.

Second, the mismatch of the demagnetization amplitude between theory and experiment is a well-known problem in ab initio spin dynamics simulations. Many existing approaches suffer from such a drawback. For example, in time-dependent Liouville equation calculations (*94*),

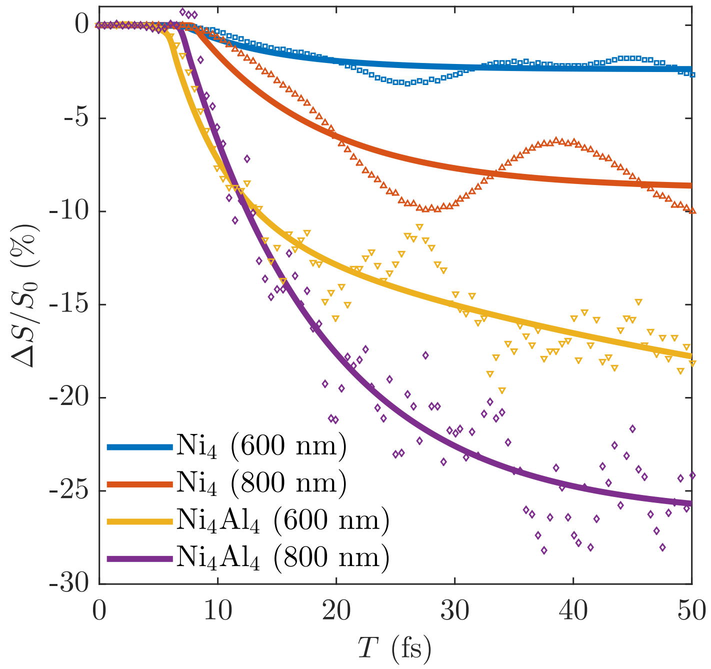

Fig. S4. Ni spin dynamics in the Ni_4 and Ni_4Al_4 slabs. Different lines represent different systems and different laser wavelengths. The y-axis value ($\Delta S/S_0$) is the total Ni spin quenching rate. Lines are the smooth fitting of data points from RT-TDDFT simulations.

the Ni demagnetization amplitude is about 1% under a laser fluence of 11.5 mJ/cm^2. In TDDFT simulations (*63*), the Ni demagnetization rate can reach 43% when a very strong laser with a fluence of 934.8 mJ/cm^2 is used. However, this fluence is much stronger than the experimental laser values and will destroy the samples. Because the demagnetization amplitude is almost proportional to the laser fluence, we can estimate the rate of these two works under 20 mJ/cm^2 used in our simulation. The corresponding values are about 2% and 1%, respectively (note that this is a very simple approximation). Our demagnetization rate (3% or 9%) is larger than these values. Nevertheless, it is still smaller than the experimental observation. Such a mismatch between theory and experiment has puzzled the field for many years.

As for FM bulk systems or isolated FM slabs (usually packed on top of an insulator substrate), there is no spin transport. The FM demagnetization is fully contributed by its own

SOC channel. Then the question arises: What causes the underestimation of the SOC rate? Zhang et al. found that the inclusion of the exchange-correlation effect can increase the 1% demagnetization in their original simulations (*94*) to around 10% (*95*). Later, Acharya et al. demonstrated that the addition of the memory effect of the electron-electron exchange-correlation interaction can further increase the demagnetization rate (*31*). In our previous work (*96*), we found that the finite-temperature spin disorder (with the inclusion of the exchange-correlation effect and the update of the electron-electron interaction) can dramatically boost the SOC demagnetization rate. These works provide insight into the mismatch problem in bulk systems.

As for the FM/NM multilayer thin film systems, many experiments (*3,32,51,59*) have found there is large spin transport from FM to NM. In the main text (Fig. 2), we prove this point and demonstrate that this large spin transport can remarkably increase the FM demagnetization rate to the scale in consistency with the experiments. This connects the ab initio simulations with the current experimental progress on FM/NM multilayer thin films, and helps us to reveal the intrinsic physics for the spin transport between FM and NM. Nevertheless, we do not include the finite-temperature spin disorder effect in our simulation, which should play a role in FM demagnetization. In order to consider a thermally disordered spin orientation, one has to use a much larger supercell with more lattice sites and random spin orientations. The corresponding computation would be much more time-consuming. In this work, we mainly focus on the exchange-induced spin transport between FM and NM, and the spin exchange constant (J_{ij}) is usually independent of spin orientations in most models (e.g., the Heisenberg model and the Landau-Lifshitz-Gilbert model). We thus do not include the spin disorder effect here.

7. Spin and charge dynamics in different layers

Fig. S5 shows the spin change of different Al layers in the Ni_4Al_{12} slab under laser excitation. The spin change of different Ni layers has been shown in Fig. 2B of the main text. They are the quantitative results of Fig. 3A of the main text. It can be seen that the amount of injected spin increases slightly with the distance to the interface. There are more spins near the Al surface than near the interface. Together with Fig. 2B of the main text, this also confirms the long-range spin transport between Ni and Al.

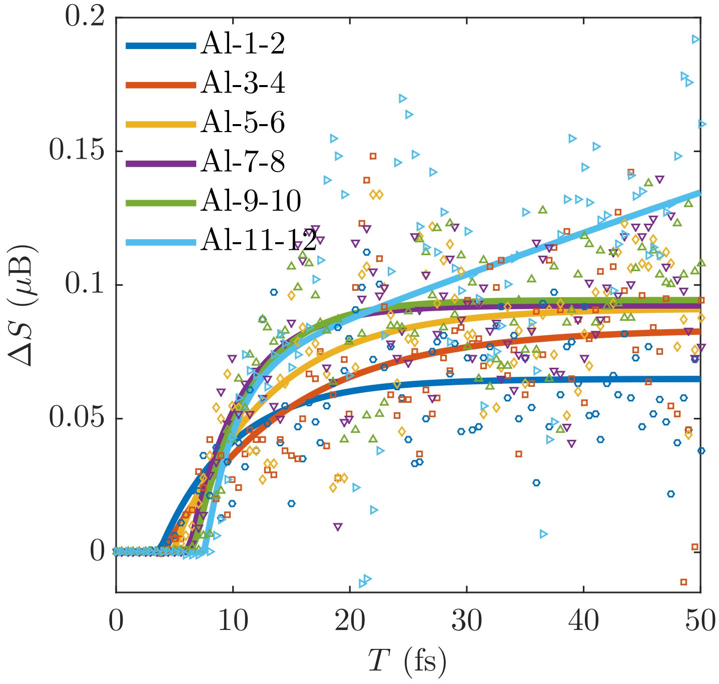

Fig. S5. Spin dynamics of different Al layers in the Ni_4Al_{12} slab. The y-axis value is the spin change amplitude (ΔS). The Al-1-2 line indicates the total spin change of the first and the second Al monolayers (i.e., interface layers in Fig. S2). Other lines are similar. This is the quantitative result of Fig. 3A in the main text. Lines are the smooth fitting of data points from RT-TDDFT simulations.

As a comparison, Fig. S6 shows the charge change of different Ni and Al layers in the Ni_4Al_{12} slab. Different from spin change, the major charge change occurs between the interface

Ni and the interface Al layers. For the layers away from the interface, the charge change is much reduced in both Ni and Al. This indicates that the charge flow is short-range and localized within the interface. Moreover, its amplitude is much smaller than the amplitude of spin change. Such a small and localized charge particle transport cannot be the reason for the large and long-range spin transport.

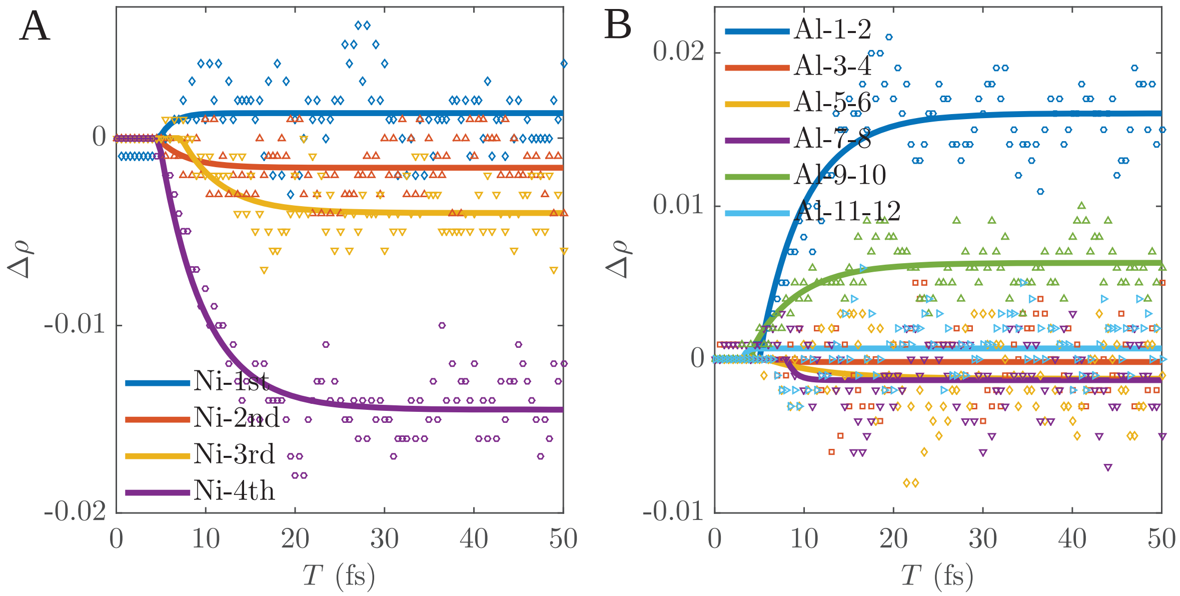

Fig. S6. Charge dynamics of different layers in the Ni_4Al_{12} slab. (**A**) Ni layers and (**B**) Al layers. The y-axis value is the charge change amplitude ($\Delta\rho$). Ni-1st is the surface Ni monolayer while Ni-4th is the interface Ni monolayer. The Al-1-2 line indicates the total charge change of the first and the second Al monolayers (see Fig. S2). Other lines are similar. Lines are the smooth fitting of data points from RT-TDDFT simulations.

Fig. S7 shows the charge change of the whole Ni film in the Ni_4Al_4, Ni_4Al_6, Ni_4Al_8, and Ni_4Al_{12} slabs. It can be seen that there is no clear relation between the amount of injected charge particles and the thickness of Al film. Charge injection does not increase with Al thickness. This is not surprising, because the charge change is localized within the interface, as shown in Fig. S6. It is mainly influenced by the interface electronic structure, while Al thickness has a small effect on the interface. Such behavior is different from spin transport (Figs. 2A and 2C

of the main text), the amount of which increases with Al film thickness. This provides another evidence for the negligible role of charge carrier transport.

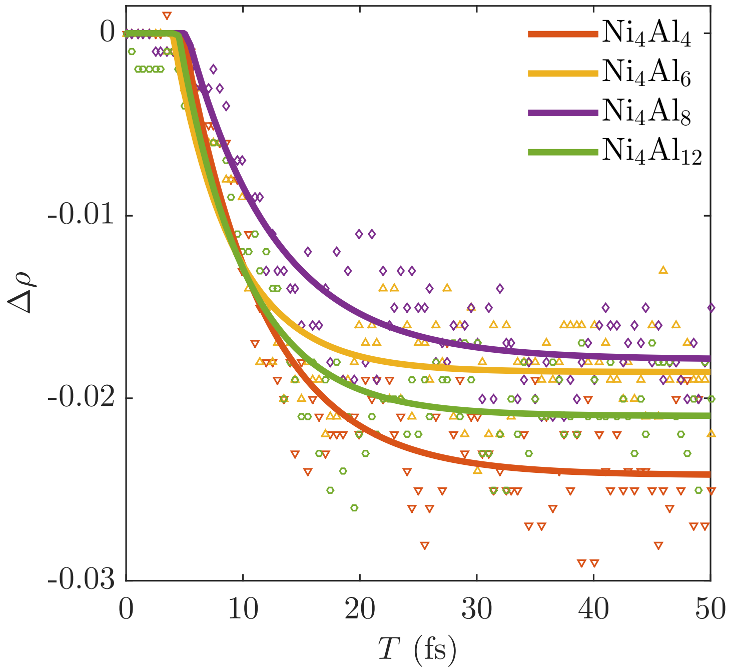

Fig. S7. Charge dynamics of the whole Ni film in different slabs. The y-axis value is the total charge change amplitude ($\Delta\rho$). Lines are the smooth fitting of data points from RT-TDDFT simulations.

8. Notes on SOC, phonon, and noncollinear magnetic moment

Fig. S8 shows the spin and charge evolution of the Ni_4Al_{12} slab when SOC is turned off. The spin dissipation channel is closed in this situation, and the spin change arises only from spin transport between Ni and Al. It can be seen that all of the Ni spin loss is fully transferred to Al (Fig. S8A), and the corresponding charge flow is about 30 times smaller than the spin flow (Fig. S8B). As has been discussed in the main text, this indicates that the charge carrier-induced

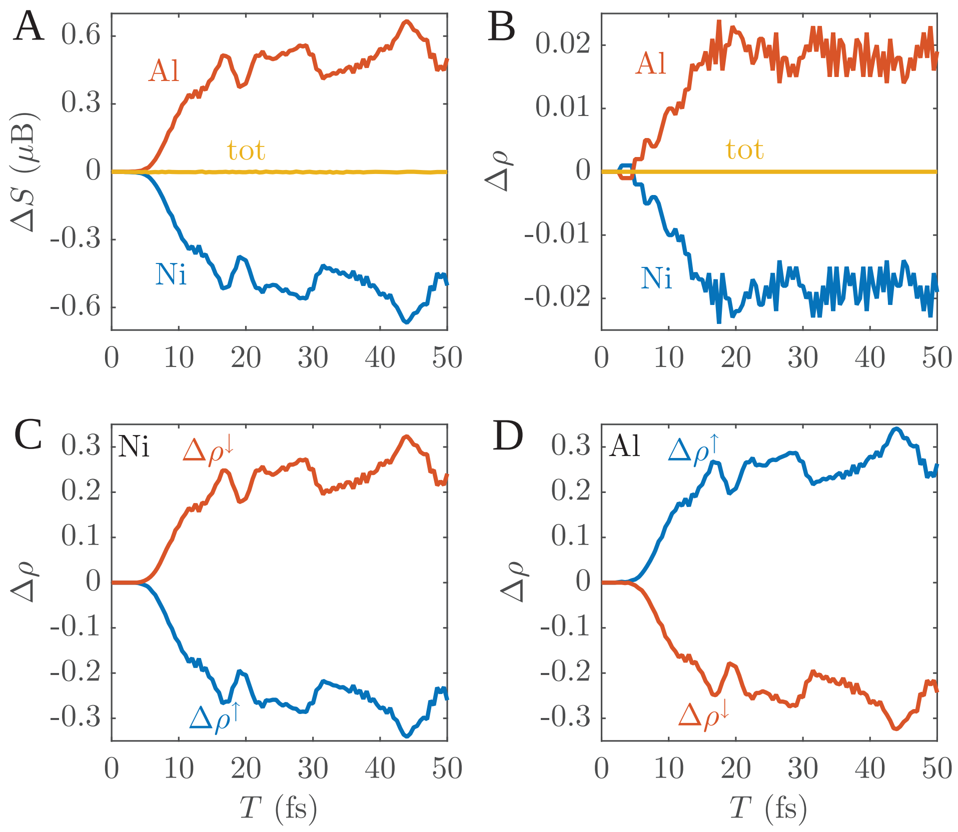

Fig. S8. Spin and charge evolution of the Ni_4Al_{12} slab when SOC is turned off. (**A** and **B**) The spin change (ΔS) and charge change ($\Delta\rho$) of Ni, Al and total. (**C** and **D**) The spin-decomposed charge change in Ni and Al.

superdiffusive spin current mechanism and the OISTR mechanism are not the reason for the large spin transport here. Figs. S8C and S8D demonstrate that spin-up charge switches to spin-down charge in Ni, and in the meantime spin-down charge switches to spin-up charge in Al. Their switching amplitudes and trajectories are exactly matched. This confirms the dominant role of *sp*–*d* spin exchange between Ni and Al.

As for the phonon, it can play two kinds of roles. First, it provides an additional angular momentum sink. The spin angular momentum can be transferred to the electronic orbital via SOC in the first step, which is further transferred to the lattice via electron-phonon coupling in

the second step. The SOC channel is necessary, and the direct angular momentum transfer from spin to lattice is negligible. In our previous work (*96*), we have studied this topic and found that the phonon can play a certain role (but not essential) in this spin relaxation process for the ferromagnetic bulk system. The case should be similar in the thin film system. Second, the lattice could affect the *sp–d* exchange coupling constant and could change the spin transport between FM and NM a little bit. In our simulations, we have studied many different types of systems. The lattices in these systems are different. For example, the relative Ni and Al atomic positions are not the same for Ni_4Al_4, Ni_6Al_6, Ni_8Al_8, Ni_4Al_6, Ni_4Al_8, and Ni_4Al_{12}. Their atomic differences are in the same order as the atomic movements of each system in the first 50 fs of RT-TDDFT simulations. Large spin transports have been observed in all these systems. Thus, we think that the atomic movements of each system (i.e., phonon) could change a little bit the spin transport amplitude, but will not change the overall spin transport picture.

As for the noncollinear magnetic moment, our Hamiltonian (Eqs. (1-3) of the main text) has included this effect and the spin is allowed to rotate in the three-dimensional space. But here we do not consider a thermally disordered spin orientation, which requires a much larger supercell with more lattice sites. In the section on the mismatch problem between theory and experiment, we discussed this problem and mentioned that such calculations are extremely time-consuming. Because we mainly focus on the exchange-induced spin transport and the spin exchange constant (J_{ij}) is usually independent of spin orientations, we do not include the disorder effect in this work.

9. Comparisons between very short laser excitations and manual excitations

In the main text, we have compared the charge dynamics and spin dynamics between very short laser excitations and manual excitations. Here, we present some related results.

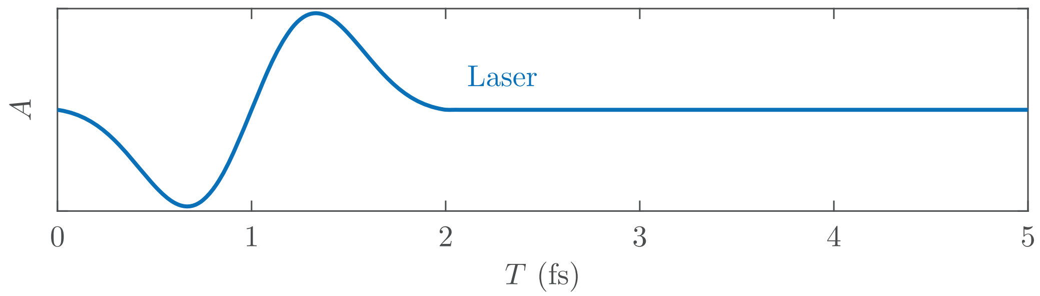

Fig. S9. The magnetic vector potential (A) of the laser with 2-fs duration.

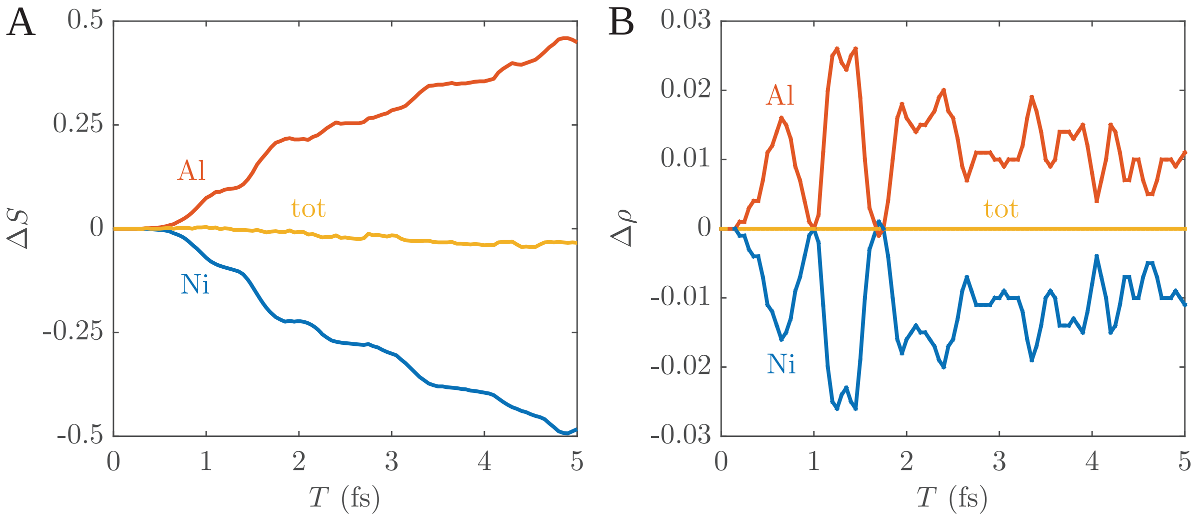

Fig. S10. Dynamics induced by the laser with 2-fs duration. (**A**) Spin change (ΔS) and (**B**) charge change ($\Delta\rho$) of the Ni_4Al_{12} slab.

Fig. S9 shows the shape of the laser with 2-fs duration and 1-fs FWHM. This laser is used to excite the Ni_4Al_{12} system. Fig. S10 shows the corresponding results regarding the spin change (ΔS) and charge change ($\Delta\rho$) of Ni, Al, and total. The comparison of the total Al charge change and spin change has been shown in Fig. 4A of the main text.

Fig. S11 shows the shape of the laser with 0.5-fs duration and 0.15-fs FWHM, and the comparison of the Al charge change and spin change of Ni_4Al_{12} excited by this laser. It demonstrates the decoupling of optical excitation and spin transport on the timescale, as well as the decoupling of charge dynamics and spin dynamics.

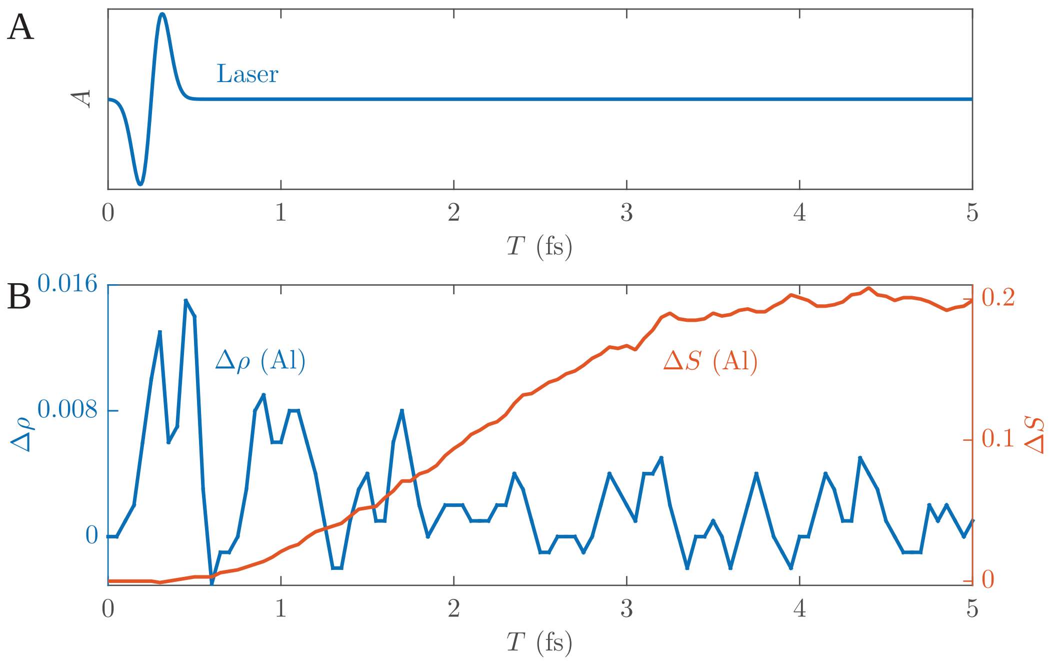

Fig. S11. Dynamics induced by the laser with 0.5-fs duration. (**A**) The magnetic vector potential (A) of the laser with 0.5-fs duration. (**B**) The Al charge change ($\Delta\rho$, the left y-axis) and spin change (ΔS, the right y-axis) of the Ni_4Al_{12} slab excited by this laser.

Fig. S12 shows three more similar simulations with the manual excitations coming from different bands and different numbers of electrons. The results are very similar to those in Fig. 4B of the main text.

10. Notes on OISTR

In the main text, we have compared our *sp–d* spin exchange mechanism with the previous OISTR mechanism. In order to avoid any misunderstanding, we provide some additional notes on OISTR here.

First, we would like to note that our descriptions about the physics of OISTR are exactly the same as those in the literature. Take Ref. 3 for Ni/Pt and Ref. 54 for CoPt as examples. Both papers are representative works of OISTR in metallic systems. Fig. 1C of Ref. 3 and Fig. 1B of

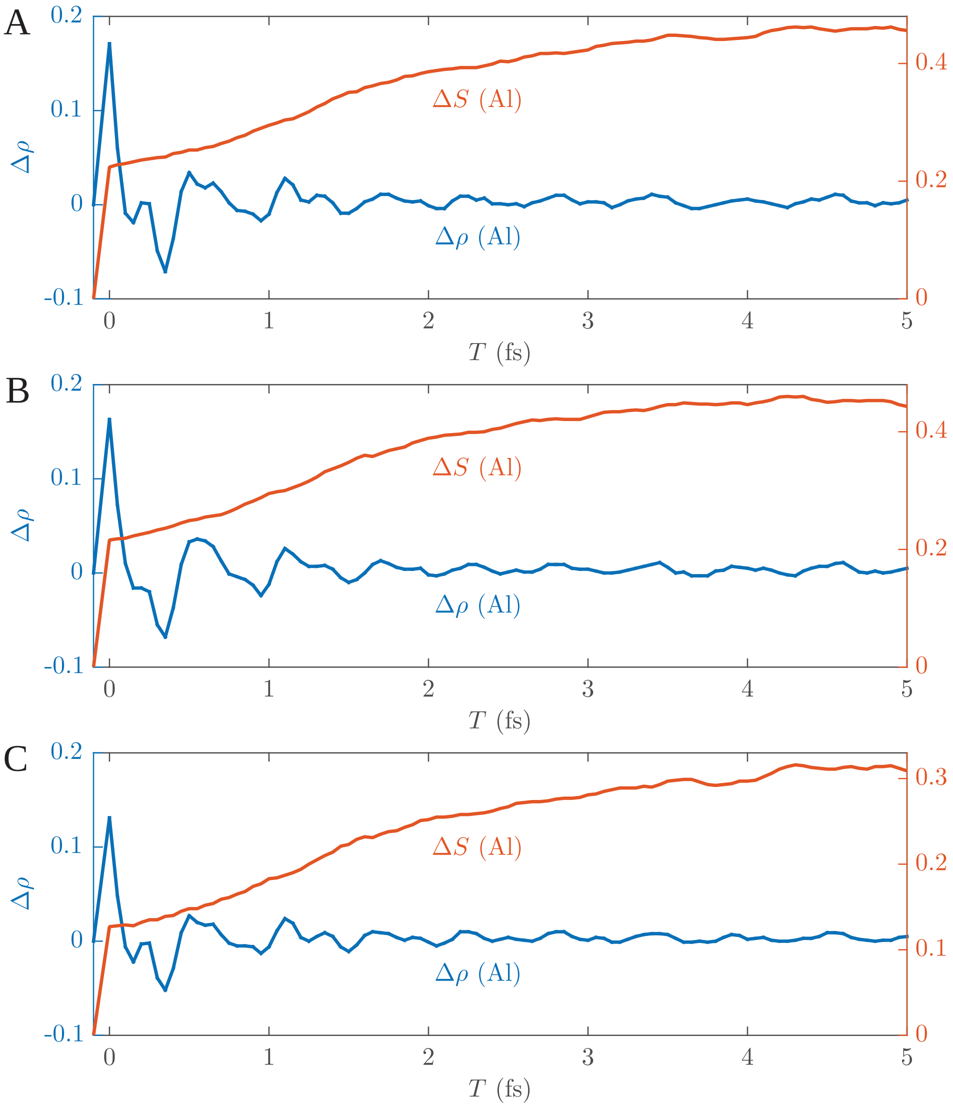

Fig. S12. Dynamics induced by manual excitation at 0 fs. (**A**, **B**, and **C**) Three different excitations on the Ni_4Al_{12} slab. The left y-axis value is the Al charge change ($\Delta\rho$), and the right y-axis value is the Al spin change (ΔS). The value below 0 fs is the referenced ground state before excitation.

Ref. 54 present a schematic illustration about how OISTR works, respectively. Both schematics and the corresponding captions demonstrate that OISTR is driven by charge carrier transport from FM to NM. Fig. 1D of Ref. 3 also illustrated a schematic figure of electron wavelet transport from Ni to Pt. Our Fig. 1B of the main text and the corresponding descriptions have

exactly the same physics as in these literatures.

Second, because OISTR is driven by charge carrier transfer, it is based on Coulomb interaction. In comparison, our *sp–d* spin exchange theory is based on exchange interaction and does not rely on charge carrier transfer. Their physics is completely different.

Third, OISTR might lead to a finally canceled charge transfer, resulting in pure spin transfer in some systems (e.g., FeNi alloy (*22*) and NiO (*52*)). The magnetic atoms of these systems are usually surrounded by other types of atoms (*52*). Nevertheless, the pure spin transfer from a canceled charge transfer is still driven by Coulomb interaction. Because of this, we performed many additional calculations (e.g., Figs. 3F−3I, Figs. 4A−4B and Figs. 5A−5B), and found much more evidence to distinguish OISTR and our *sp–d* spin exchange picture, as discussed in the main text.

11. Different region excitations

In Figs. 5A and 5B of the main text, we have compared the spin dynamics when the laser is shone on the different regions of the Ni_4Al_{12} slab. Fig. S13 shows all the spin curves (9 in total) of Ni, Al, and total under three different excitations. In order to understand these results in more detail, we have summarized the comparisons of different excitations in Table S1 and explained them as follows:

(1) In the case of shining the laser on both Ni and Al regions (i.e., "excite-both"), Ni intrinsic spin dissipation exists due to its strong SOC (while the SOC of Al is much weaker and its intrinsic spin dissipation is negligible in all cases). Because the light excitation creates more free carriers and larger empty "room" in both Ni and Al for *sp–d* spin exchange, there also exists large spin transport from Ni to Al. Note that the Ni spin change is contributed by both spin dissipation and spin transport, while the Al spin change is contributed only by spin transport. The total spin change of the whole system arises only from Ni spin dissipation because spin

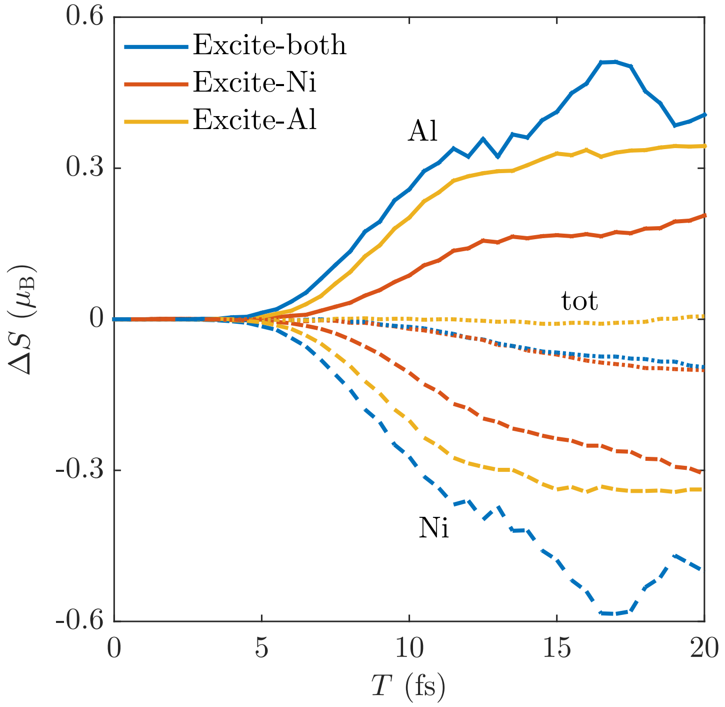

Fig. S13. Spin evolution of different regions of Ni_4Al_{12} under different excitations. The solid lines are the Al spins. The dashed lines are the Ni spins. The dotted lines are the total spins.

Table S1. Spin dynamics under three different types of laser excitation.

	Excite both	Excite Ni	Excite Al
SOC-induced Ni spin dissipation	Yes	Yes	Almost zero
Spin transport from Ni to Al	Larger	Small	Large
Ni spin change (contributed by spin dissipation and spin transport)	Large	Small	Small
Al spin change (contributed only by spin transport)	Larger	Small	Large
Total spin change (contributed only by Ni spin dissipation)	Yes	Yes	Almost zero

transport only redistributes the spin within the whole system. As a result, all of the Ni spin, Al spin, and total spin have a large change in the "excite-both" case. This case is very common in the experiments of multilayer FM/NM thin films, where the incident light can penetrate into both FM and NM.

(2) In the case of shining the laser only on the Ni region (i.e., "excite-Ni"), Ni intrinsic spin dissipation still exists. However, the spin transport from Ni to Al is dramatically reduced, because the carrier excitation in Al is strongly restricted and there is no large amount of free carriers and no large empty "room" to involve in the spin exchange with Ni. Nevertheless, the amplitude of this spin transport is not zero, because there is a certain amount of delocalized thermal carriers in Al at room temperature. Compared to the "excite-both" case, the Ni spin change and Al spin change here are smaller, but the total spin change is close. In experiments, this case is somewhat similar to light excitation in a very thick FM bulk system on the NM substrate or an FM/insulator system, where only FM carriers are optically excited.

(3) In the case of shining the laser only on the Al region (i.e., "excite-Al"), Ni intrinsic spin dissipation is almost zero because Ni carrier excitation is strongly restricted. The spin exchange between Ni and Al is also reduced compared to the "excite-both" case, but is stronger than the "excite-Ni" case. It implies that Al excitation is more important than Ni excitation in spin transport. The Ni spin change and Al spin change here are smaller than those in the "excite-both" case, and the total spin change is almost zero. In experiments, this case is like a NM covering on top of FM. Most of the incident light is absorbed in the NM and cannot enter into the FM. Such NM/FM/substrate systems with a front laser shining or FM/NM/insulator systems with a back laser shining have also been investigated by many experiments (*32, 59*).

12. Different energy excitations

In Figs. 5C and 5D of the main text, we have compared the spin dynamics when the Ni_4Al_{12} slab is excited by the lasers with different fluences. Fig. S14 shows all the spin curves (9 in total) of Ni, Al and total under three different excitations.

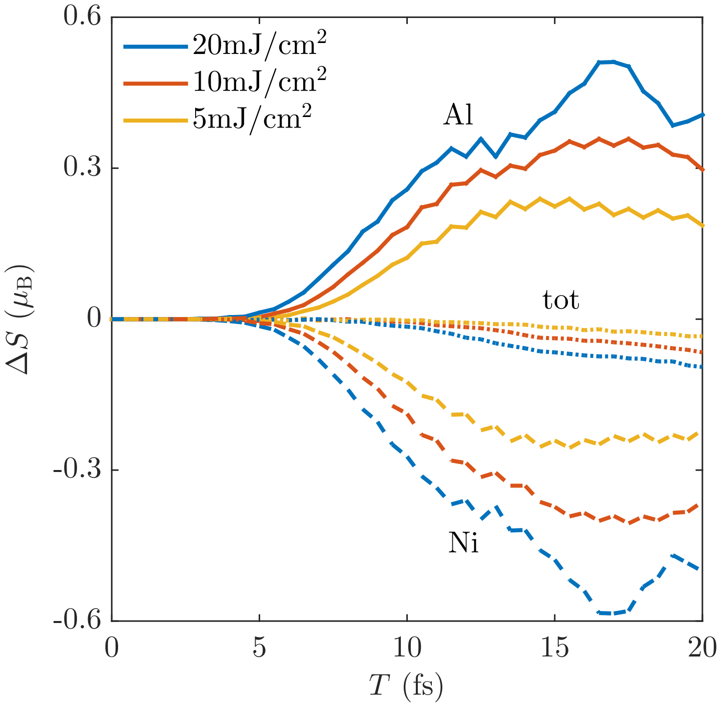

Fig. S14. Spin evolution of different regions of Ni_4Al_{12} under different excitations. The solid lines are the Al spins. The dashed lines are the Ni spins. The dotted lines are the total spins.

13. Dependence on the slab thickness

In Figs. 6A and 6B of the main text, we have shown that the Ni demagnetization rate has no obvious decay with the increase of the slab thickness, and the Ni layers far away from the interface still have large demagnetization in the Ni_8Al_8 slab. Fig. S15 further provides the corresponding data for the spin change of different Ni layers in the Ni_4Al_4 and Ni_6Al_6 slabs.

Like the results of Ni_8Al_8, all of their Ni layers have large demagnetization rates, though with some fluctuations between different layers. These results give a sense of what the spin dynamics will be like in thicker systems.

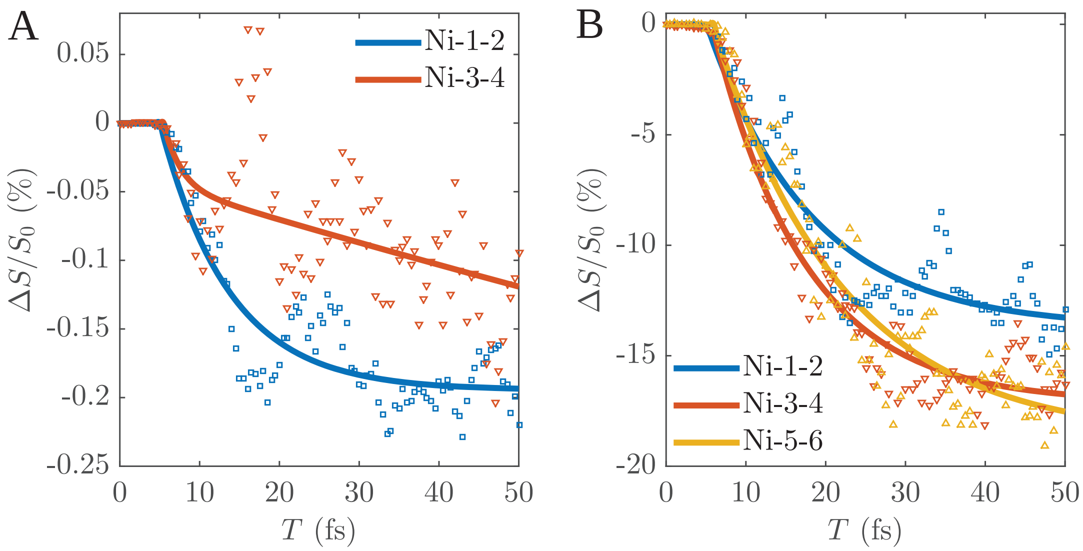

Fig. S15. Ni spin dynamics. (**A**) The Ni_4Al_4 slab and (**B**) the Ni_6Al_6 slab. The y-axis value is the spin quenching rate ($\Delta S/S_0$) of different Ni layers. Ni-1-2 represent the two surface Ni layers. Ni-3-4 represent the two interface layers in Ni_4Al_4 and Ni-5-6 represent the two interface layers in Ni_6Al_6. Lines are the smooth fitting of data points from RT-TDDFT simulations.

We would like to note that the exchange-induced spin transport takes place almost instantaneously with light shining. The light excitation (at FM, NM, or both) generates more carriers and also increases the carrier temperatures. This will immediately break the initial spin exchange equilibrium and drive the spin transfer from *d* states to *sp* states. Once the carrier excitation accumulates to a certain amount, the *sp*–*d* spin exchange process becomes visible, as at about 10 fs in our simulations. If the light is shone on the FM side, the relatively large spin transport should happen almost at the same time, irrespective of the NM slab thickness. This is because the amounts of carrier excitations around the FM/NM interface are almost the same for different NM slab thicknesses. However, if the FM slab becomes much thicker, there should be

a varying time offset with the FM thickness. This is because the light absorption decays with the FM depth, and the amount of interface excitations becomes smaller in a thicker FM slab. The large spin transport will thus be delayed in this case. In our simulations for Ni_4Al_4, Ni_6Al_6, and Ni_8Al_8, their thicknesses are much smaller than the light adsorption constant. We thus do not consider the effect of the spatial dependence of the light field. As a result, the start time of spin transport looks almost the same for Ni_4Al_4, Ni_6Al_6, and Ni_8Al_8 in our simulations.

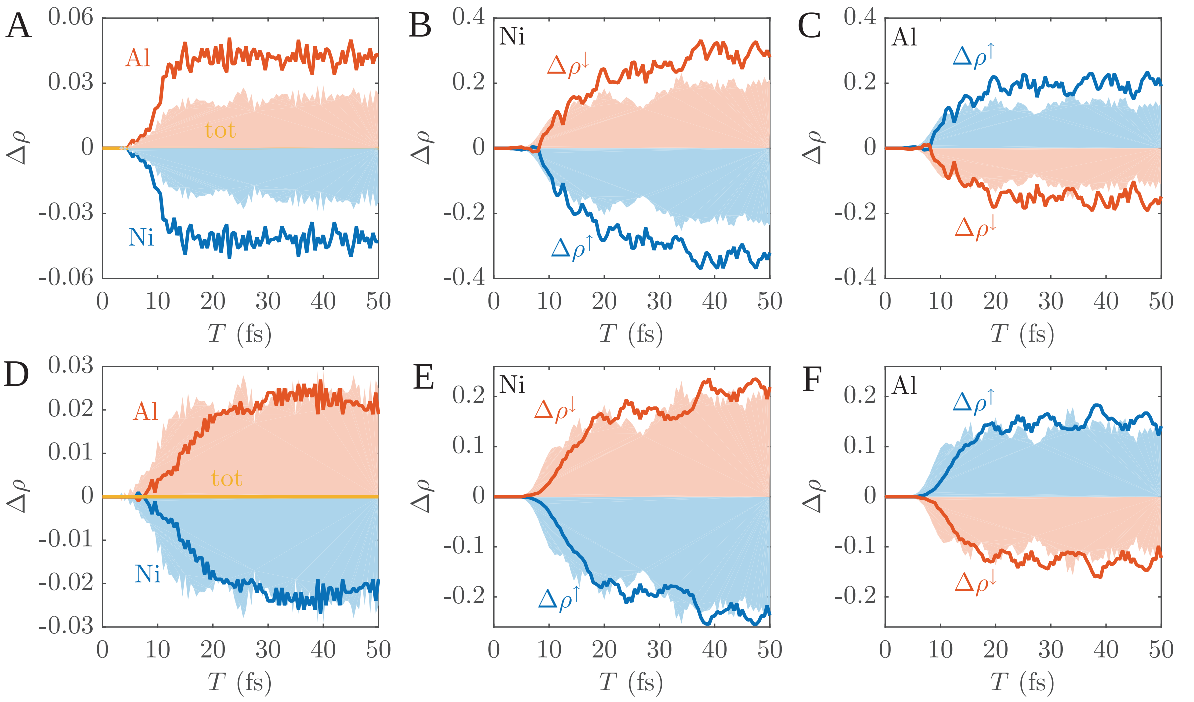

Fig. S16. Charge dynamics of the Ni_4Al_4 slab. (**A** to **C**) The case when the laser wavelength is changed to 800 nm and (**D** to **F**) the case when the laser duration is changed to 30 fs (with 12-fs FWHM), compared with the original simulation (shaded areas) with 600-nm wavelength and 20-fs duration (with 8-fs FWHM). All other laser parameters in the two simulations are kept the same as in the original simulation. The y-axis value in **A** and **D** is the charge change ($\Delta\rho$) of Ni, Al, and total. The y-axis value in **B**, **C**, **E**, and **F** is the spin-decomposed charge change in Ni and Al.

14. Dependence on the laser wavelength and width

In Figs. 6D and 6E of the main text, we have shown the large spin transport between Ni and Al under different laser wavelengths and widths. Fig. S16 provides the corresponding total charge change and spin-decomposed charge change. It can be seen that the charge flow from Ni to Al is very small. In both of the two different laser excitations, spin-up charge particles transfer to spin-down charge particles in Ni, and in the meantime Al has the inverse transfer from spin-down to spin-up. This demonstrates the spin exchange process.

15. More comparisons with experiments

In addition to the experimental work discussed in the main text, *sp–d* exchange-induced spin transport theory can be used in the interpretation of more experiments. Here we have provided several others with different structures, as below.

(1) In Ref. 11, Malinowski et al. demonstrated that interlayer transfer of spin angular momentum in [Co-Pt]/Ru/[Co-Pt] multilayer thin films speeds up the demagnetization process. It was shown that the demagnetization rate when the magnetic moments of the left Co-Pt layer and the right Co-Pt layer are antiparallel is much larger than the rate when their magnetic moments are parallel. This result can also be explained by our *sp–d* spin transport theory, as for the Ni/Al, Ni/Pt, and Fe/Au systems in the main text. When the magnetic moments of the two terminals are parallel, the spin flow from left to right and the one from right to left cancel each other out. In contrast, when their magnetic moments are antiparallel, the two spin flows will add up together and speed up the demagnetization in each terminal.

(2) In Ref. 80, Alekhin et al. designed a Fe(14.6 nm)/Au(49.4 nm)/Fe(12.7 nm) thin film. They investigated four different cases by controlling the light pumping position and the Fe magnetic moment directions. This includes pumping the left Fe layer when the left Fe spin and

the right Fe spin are (A) parallel, (B) antiparallel, and (C) perpendicular, as well as (D) pumping the right Fe layer when the left Fe spin and the right Fe spin are parallel. All the probing was performed on the right Fe layer. In all four cases, it was found that the spin is transported from the pumping Fe layer to the other Fe layer, and the magnetic moment direction of spin flow stays the same as that of the pumping Fe layer. These phenomena are in accordance with our *sp–d* spin exchange theory. First, let us take cases A, B, and C as examples. The pumping light excites the left Fe layer and part of the Au layer, which increases the density and temperature of free *sp* carriers. This breaks the initial equilibrium state of *sp–d* spin exchange and causes the pure spin transport from the left Fe-*d* to Au-*sp*. The Au-*sp* spin flow moves further into the right Fe layer through the same *sp–d* spin exchange interaction. Because angular momentum is conserved during *sp–d* spin exchange, the Au-*sp* magnetic moment keeps the same polarization as the source Fe-*d* magnetic moment. Such polarized spin flow causes the right Fe spin enhancement in case A, reduction in case B, and precession in case C.

Next, let us compare cases A and D, which can help us estimate the contribution of spin dissipation and spin transport. In case A, the $\Delta E_m/E_m$ rate (a rate related to spin change) of the right Fe layer is about 7%, which is caused only by the spin transport from the left Fe layer and Au layer. Because there should be some injected spins remaining in Au, the total spin transport rate will be over 7%. In case D, the measured $\Delta E_m/E_m$ rate is about 12%, which is caused by both spin dissipation and spin transport. Because the pumping light is the same for the two cases and the thicknesses of the left Fe layer and the right Fe layer are close, we can reasonably assume that the spin transport rate is close in both cases. Consequently, in case D, spin transport contributes more than 7% and spin dissipation contributes less than 5%. This supports the importance of *sp–d* spin transport in the demagnetization process.

(3) In Ref. 50, Rudolf et al. studied the laser-induced spin dynamics in the Al(3 nm)/Ni(5 nm)/Ru(1.5 nm)/Fe(4 nm)/Ta(3 nm) multilayer thin films. Like the above two experiments,

Fe spin enhancement and reduction were observed when Ni spin and Fe spin are parallel and antiparallel, respectively. The authors found spin flow from Ni to the right Ru, the right Fe, and the left Al. The spin transport from Ni to Al accords with our simulations in the main text. As for the spin transport from Ni to Ru and Fe, it is very similar to the above Fe/Au/Fe sample. Ru has the same function as Au, and its *sp* electrons act as a shuttle to transfer the spin.

We would like to note that some experimental samples have a very thin capping layer or seeding layer in the main metal layers. For example, the Au/Ni/Pt/Al sample in Ref. 32 contains very thin Pt layers ($\sim$2.5 nm) as the seeding layer or capping layer. As simulated in Fig. 6F of the main text, strong *sp–d* spin exchange coupling exists between Ni and Pt, and the *sp* state of Pt is a good spin conductor. Thus, these structures do not change the underlying *sp–d* spin exchange physics.

16. Supplementary movie

Movie S1. Spin density evolution of the Ni_4Al_{12} slab. The time resolution is 2 frames per fs. The spin density color bar is the same as that in Fig. 3A of the main text.

REFERENCES AND NOTES

1. A. V. Kimel, M. Li, Writing magnetic memory with ultrashort light pulses. *Nat. Rev. Mater.* **4**, 189–200 (2019).

2. C. Dornes, Y. Acremann, M. Savoini, M. Kubli, M. J. Neugebauer, E. Abreu, L. Huber, G. Lantz, C. A. Vaz, H. Lemke, E. M. Bothschafter, M. Porer, V. Esposito, L. Rettig, M. Buzzi, A. Alberca, Y. W. Windsor, P. Beaud, U. Staub, D. Zhu, S. Song, J. M. Glownia, S. L. Johnson, The ultrafast Einstein–de Haas effect. *Nature* **565**, 209–212 (2019).

3. F. Siegrist, J. A. Gessner, M. Ossiander, C. Denker, Y.-P. Chang, M. C. Schröder, A. Guggenmos, Y. Cui, J. Walowski, U. Martens, J. K. Dewhurst, U. Kleineberg, M. Münzenberg, S. Sharma, M. Schultze, Light-wave dynamic control of magnetism. *Nature* **571**, 240–244 (2019).

4. A. Stupakiewicz, K. Szerenos, D. Afanasiev, A. Kirilyuk, A. Kimel, Ultrafast nonthermal photo-magnetic recording in a transparent medium. *Nature* **542**, 71–74 (2017).

5. Y. Windsor, S.-E. Lee, D. Zahn, V. Borisov, D. Thonig, K. Kliemt, A. Ernst, C. Schüßler-Langeheine, N. Pontius, U. Staub, C. Krellner, D. Vyalikh, O. Eriksson, L. Rettig, Exchange scaling of ultrafast angular momentum transfer in 4f antiferromagnets. *Nat. Mater.* **21**, 514–517 (2022).

6. G. Zhang, Y. Bai, M. Si, T. F. George, First-principles insight into all-optical spin switching in the half-metallic Heusler ferrimagnet Mn_2RuGa. *Phys. Rev. B* **105**, 054431 (2022).

7. B. Gu, D. Keefer, F. Aleotti, A. Nenov, M. Garavelli, S. Mukamel, Photoisomerization transition state manipulation by entangled two-photon absorption. *Proc. Natl. Acad. Sci. U.S.A.* **118**, e2116868118 (2021).

8. E. Golias, I. Kumberg, I. Gelen, S. Thakur, J. Gördes, R. Hosseinifar, Q. Guillet, J. K. Dewhurst, S. Sharma, C. Schüßler-Langeheine, N. Pontius, W. Kuch, Ultrafast optically induced ferromagnetic state in an elemental antiferromagnet. *Phys. Rev. Lett.* **126**, 107202 (2021).

9. S. Tauchert, M. Volkov, D. Ehberger, D. Kazenwadel, M. Evers, H. Lange, A. Donges, A. Book, W. Kreuzpaintner, U. Nowak, P. Baum, Polarized phonons carry angular momentum in ultrafast demagnetization. *Nature* **602**, 73–77 (2022).

10. A. Kirilyuk, A. V. Kimel, T. Rasing, Ultrafast optical manipulation of magnetic order. *Rev. Mod. Phys.* **82**, 2731–2784 (2010).

11. G. Malinowski, F. Dalla Longa, J. Rietjens, P. Paluskar, R. Huijink, H. Swagten, B. Koopmans, Control of speed and efficiency of ultrafast demagnetization by direct transfer of spin angular momentum. *Nat. Phys.* **4**, 855–858 (2008).

12. C. Boeglin, E. Beaurepaire, V. Halté, V. López-Flores, C. Stamm, N. Pontius, H. Dürr, J.-Y. Bigot, Distinguishing the ultrafast dynamics of spin and orbital moments in solids. *Nature* **465**, 458–461 (2010).

13. E. Beaurepaire, J.-C. Merle, A. Daunois, J.-Y. Bigot, Ultrafast spin dynamics in ferromagnetic nickel. *Phys. Rev. Lett.* **76**, 4250–4253 (1996).

14. A. Melnikov, I. Razdolski, T. O. Wehling, E. T. Papaioannou, V. Roddatis, P. Fumagalli, O. Aktsipetrov, A. I. Lichtenstein, U. Bovensiepen, Ultrafast transport of laser-excited spin-polarized carriers in Au/Fe/MgO(001). *Phys. Rev. Lett.* **107**, 076601 (2011).

15. J.-Y. Bigot, M. Vomir, E. Beaurepaire, Coherent ultrafast magnetism induced by femtosecond laser pulses. *Nat. Phys.* **5**, 515–520 (2009).

16. C. Stamm, T. Kachel, N. Pontius, R. Mitzner, T. Quast, K. Holldack, S. Khan, C. Lupulescu, E. F. Aziz, M. Wietstruk, H. A. Dürr, W. Eberhardt, Femtosecond modification of electron localization and transfer of angular momentum in nickel. *Nat. Mater.* **6**, 740–743 (2007).

17. A. Goris, K. Döbrich, I. Panzer, A. Schmidt, M. Donath, M. Weinelt, Role of spin-flip exchange scattering for hot-electron lifetimes in cobalt. *Phys. Rev. Lett.* **107**, 026601 (2011).

18. L.-O. Chan, E. Turgut, C. A. Teale, H. C. Kapteyn, M. M. Murnane, S. Mathias, M. Aeschlimann, C. M. Schneider, J. M. Shaw, H. T. Nembach, T. J. Silva, Ultrafast

demagnetization measurements using extreme ultraviolet light: Comparison of electronic and magnetic contributions. *Phys. Rev. X* **2**, 011005 (2012).

19. T. Roth, A. Schellekens, S. Alebrand, O. Schmitt, D. Steil, B. Koopmans, M. Cinchetti, M. Aeschlimann, Temperature dependence of laser-induced demagnetization in Ni: A key for identifying the underlying mechanism. *Phys. Rev. X* **2**, 021006 (2012).

20. W. You, P. Tengdin, C. Chen, X. Shi, D. Zusin, Y. Zhang, C. Gentry, A. Blonsky, M. Keller, P. M. Oppeneer, H. Kapteyn, Z. Tao, M. Murnane, Revealing the nature of the ultrafast magnetic phase transition in Ni by correlating extreme ultraviolet magneto-optic and photoemission spectroscopies. *Phys. Rev. Lett.* **121**, 077204 (2018).

21. G. Zhang, M. Murakami, All-optical spin switching under different spin configurations. *J. Phys. Condens. Matter* **31**, 345802 (2019).

22. M. Hofherr, S. Häuser, J. Dewhurst, P. Tengdin, S. Sakshath, H. Nembach, S. Weber, J. Shaw, T. Silva, H. Kapteyn, M. Cinchetti, B. Rethfeld, M. M. Murnane, D. Steil, B. Stadtmüller, S. Sharma, M. Aeschlimann, S. Mathias, Ultrafast optically induced spin transfer in ferromagnetic alloys. *Sci. Adv.* **6**, eaay8717 (2020).

23. M. Beens, R. A. Duine, B. Koopmans, Modeling ultrafast demagnetization and spin transport: The interplay of spin-polarized electrons and thermal magnons. *Phys. Rev. B* **105**, 144420 (2022).

24. M. Schneider, B. Pfau, C. M. Günther, C. von Korff Schmising, D. Weder, J. Geilhufe, J. Perron, F. Capotondi, E. Pedersoli, M. Manfredda, M. Hennecke, B. Vodungbo, J. Lüning, S. Eisebitt, Ultrafast demagnetization dominates fluence dependence of magnetic scattering at Co *M* edges. *Phys. Rev. Lett.* **125**, 127201 (2020).

25. F. Hellman, A. Hoffmann, Y. Tserkovnyak, G. S. D. Beach, E. E. Fullerton, C. Leighton, A. H. MacDonald, D. C. Ralph, D. A. Arena, H. A. Dürr, P. Fischer, J. Grollier, J. P. Heremans, T. Jungwirth, A. V. Kimel, B. Koopmans, I. N. Krivorotov, S. J. May, A. K. Petford-Long, J. M. Rondinelli, N. Samarth, I. K. Schuller, A. N. Slavin, M. D. Stiles, O.

Tchernyshyov, A. Thiaville, B. L. Zink, Interface-induced phenomena in magnetism. *Rev. Mod. Phys.* **89**, 025006 (2017).

26. C.-H. Lambert, S. Mangin, B. C. S. Varaprasad, Y. Takahashi, M. Hehn, M. Cinchetti, G. Malinowski, K. Hono, Y. Fainman, M. Aeschlimann, E. E. Fullerton, All-optical control of ferromagnetic thin films and nanostructures. *Science* **345**, 1337–1340 (2014).

27. T. Huisman, R. Mikhaylovskiy, J. Costa, F. Freimuth, E. Paz, J. Ventura, P. Freitas, S. Blügel, Y. Mokrousov, T. Rasing, A. V. Kimel, Femtosecond control of electric currents in metallic ferromagnetic heterostructures. *Nat. Nanotechnol.* **11**, 455–458 (2016).

28. A. Schellekens, K. Kuiper, R. De Wit, B. Koopmans, Ultrafast spin-transfer torque driven by femtosecond pulsed-laser excitation. *Nat. Commun.* **5**, 4333 (2014).

29. A. L. Chekhov, Y. Behovits, J. J. F. Heitz, C. Denker, D. A. Reiss, M. Wolf, M. Weinelt, P. W. Brouwer, M. Münzenberg, T. Kampfrath, Ultrafast demagnetization of iron induced by optical versus terahertz pulses. *Phys. Rev. X* **11**, 041055 (2021).

30. G. Zhang, W. Hübner, G. Lefkidis, Y. Bai, T. F. George, Paradigm of the time-resolved magneto-optical Kerr effect for femtosecond magnetism. *Nat. Phys.* **5**, 499–502 (2009).

31. S. R. Acharya, V. Turkowski, G. Zhang, T. S. Rahman, Ultrafast electron correlations and memory effects at work: Femtosecond demagnetization in Ni. *Phys. Rev. Lett.* **125**, 017202 (2020).

32. A. Eschenlohr, M. Battiato, P. Maldonado, N. Pontius, T. Kachel, K. Holldack, R. Mitzner, A. Föhlisch, P. M. Oppeneer, C. Stamm, Ultrafast spin transport as key to femtosecond demagnetization. *Nat. Mater.* **12**, 332–336 (2013).

33. B. Koopmans, G. Malinowski, F. Dalla Longa, D. Steiauf, M. Fähnle, T. Roth, M. Cinchetti, M. Aeschlimann, Explaining the paradoxical diversity of ultrafast laser-induced demagnetization. *Nat. Mater.* **9**, 259–265 (2010).

34. R. F. Evans, W. J. Fan, P. Chureemart, T. A. Ostler, M. O. Ellis, R. W. Chantrell, Atomistic spin model simulations of magnetic nanomaterials. *J. Phys. Condens. Matter* **26**, 103202 (2014).

35. S. Eich, M. Plötzing, M. Rollinger, S. Emmerich, R. Adam, C. Chen, H. C. Kapteyn, M. M. Murnane, L. Plucinski, D. Steil, B. Stadtmüller, M. Cinchetti, M. Aeschlimann, C. M. Schneider, S. Mathias, Band structure evolution during the ultrafast ferromagnetic-paramagnetic phase transition in cobalt. *Sci. Adv.* **3**, e1602094 (2017).

36. M. Berritta, R. Mondal, K. Carva, P. M. Oppeneer, Ab initio theory of coherent laser-induced magnetization in metals. *Phys. Rev. Lett.* **117**, 137203 (2016).

37. V. Shokeen, M. S. Piaia, J.-Y. Bigot, T. Müller, P. Elliott, J. Dewhurst, S. Sharma, E. Gross, Spin flips versus spin transport in nonthermal electrons excited by ultrashort optical pulses in transition metals. *Phys. Rev. Lett.* **119**, 107203 (2017).

38. W. Töws, G. Pastor, Many-body theory of ultrafast demagnetization and angular momentum transfer in ferromagnetic transition metals. *Phys. Rev. Lett.* **115**, 217204 (2015).

39. G. Zhang, Y. Bai, T. F. George, Ultrafast reduction of exchange splitting in ferromagnetic nickel. *J. Phys. Condens. Matter* **28**, 236004 (2016).

40. J. Chureemart, R. Cuadrado, P. Chureemart, R. Chantrell, Multiscale modeling of spin transport across a diffuse interface. *J. Magn. Magn. Mater.* **443**, 287–292 (2017).

41. K. C. Kuiper, T. Roth, A. J. Schellekens, O. Schmitt, B. Koopmans, M. Cinchetti, M. Aeschlimann, Spin-orbit enhanced demagnetization rate in Co/Pt-multilayers. *Appl. Phys. Lett.* **105**, 202402 (2014).

42. D. Steil, S. Alebrand, T. Roth, M. Krauß, T. Kubota, M. Oogane, Y. Ando, H. C. Schneider, M. Aeschlimann, M. Cinchetti, Band-structure-dependent demagnetization in the heusler alloy $Co_2Mn_{1-x}Fe_xSi$. *Phys. Rev. Lett.* **105**, 217202 (2010).

43. B. Koopmans, J. Ruigrok, F. Dalla Longa, W. De Jonge, Unifying ultrafast magnetization dynamics. *Phys. Rev. Lett.* **95**, 267207 (2005).

44. K. Carva, M. Battiato, P. M. Oppeneer, Ab initio investigation of the Elliott-Yafet electron-phonon mechanism in laser-induced ultrafast demagnetization. *Phys. Rev. Lett.* **107**, 207201 (2011).

45. G. Zhang, W. Hübner, Laser-induced ultrafast demagnetization in ferromagnetic metals. *Phys. Rev. Lett.* **85**, 3025–3028 (2000).

46. J. Simoni, S. Sanvito, Conservation of angular momentum in ultrafast spin dynamics. *Phys. Rev. B* **105**, 104437 (2022).

47. S. Essert, H. C. Schneider, Electron-phonon scattering dynamics in ferromagnetic metals and their influence on ultrafast demagnetization processes. *Phys. Rev. B* **84**, 224405 (2011).

48. T. F. Nova, A. Cartella, A. Cantaluppi, M. Först, D. Bossini, R. Mikhaylovskiy, A. Kimel, R. Merlin, A. Cavalleri, An effective magnetic field from optically driven phonons. *Nat. Phys.* **13**, 132–136 (2017).

49. M. Battiato, K. Carva, P. M. Oppeneer, Superdiffusive spin transport as a mechanism of ultrafast demagnetization. *Phys. Rev. Lett.* **105**, 027203 (2010).

50. D. Rudolf, L.-O. Chan, M. Battiato, R. Adam, J. M. Shaw, E. Turgut, P. Maldonado, S. Mathias, P. Grychtol, H. T. Nembach, T. J. Silva, M. Aeschlimann, H. C. Kapteyn, M. M. Murnane, C. M. Schneider, P. M. Oppeneer, Ultrafast magnetization enhancement in metallic multilayers driven by superdiffusive spin current. *Nat. Commun.* **3**, 1037 (2012).

51. E. Turgut, J. M. Shaw, P. Grychtol, H. T. Nembach, D. Rudolf, R. Adam, M. Aeschlimann, C. M. Schneider, T. J. Silva, M. M. Murnane, H. C. Kapteyn, S. Mathias, Controlling the competition between optically induced ultrafast spin-flip scattering and spin transport in magnetic multilayers. *Phys. Rev. Lett.* **110**, 197201 (2013).

52. J. K. Dewhurst, P. Elliott, S. Shallcross, E. K. Gross, S. Sharma, Laser-induced intersite spin transfer. *Nano Lett.* **18**, 1842–1848 (2018).

53. J. Chen, U. Bovensiepen, A. Eschenlohr, T. Müller, P. Elliott, E. Gross, J. Dewhurst, S. Sharma, Competing spin transfer and dissipation at Co/Cu(001) interfaces on femtosecond timescales. *Phys. Rev. Lett.* **122**, 067202 (2019).

54. F. Willems, C. von Korff Schmising, C. Strüber, D. Schick, D. W. Engel, J. Dewhurst, P. Elliott, S. Sharma, S. Eisebitt, Optical inter-site spin transfer probed by energy and spin-resolved transient absorption spectroscopy. *Nat. Commun.* **11**, 871 (2020).

55. J. Liu, C. Li, W. Jin, G. Lefkidis, W. Hübner, Long-distance ultrafast spin transfer over a zigzag carbon chain structure. *Phys. Rev. Lett.* **126**, 037402 (2021).

56. A. R. Khorsand, M. Savoini, A. Kirilyuk, T. Rasing, Optical excitation of thin magnetic layers in multilayer structures. *Nat. Mater.* **13**, 101–102 (2014).

57. A. J. Schellekens, W. Verhoeven, T. N. Vader, B. Koopmans, Investigating the contribution of superdiffusive transport to ultrafast demagnetization of ferromagnetic thin films. *Appl. Phys. Lett.* **102**, 252408 (2013).

58. A. B. Schmidt, M. Pickel, M. Wiemhöfer, M. Donath, M. Weinelt, Spin-dependent electron dynamics in front of a ferromagnetic surface. *Phys. Rev. Lett.* **95**, 107402 (2005).

59. N. Bergeard, M. Hehn, S. Mangin, G. Lengaigne, F. Montaigne, M. Lalieu, B. Koopmans, G. Malinowski, Hot-electron-induced ultrafast demagnetization in Co/Pt multilayers. *Phys. Rev. Lett.* **117**, 147203 (2016).

60. J. Wang, C. Sun, J. Kono, A. Oiwa, H. Munekata, Ł. Cywiński, L. J. Sham, Ultrafast quenching of ferromagnetism in InMnAs induced by intense laser irradiation. *Phys. Rev. Lett.* **95**, 167401 (2005).

61. Ł. Cywiński, L. Sham, Ultrafast demagnetization in the *sp*−*d* model: A theoretical study. *Phys. Rev. B* **76**, 045205 (2007).

62. Z. Wang, S.-S. Li, L.-W. Wang, Efficient real-time time-dependent density functional theory method and its application to a collision of an ion with a 2D material. *Phys. Rev. Lett.* **114**, 063004 (2015).

63. K. Krieger, J. K. Dewhurst, P. Elliott, S. Sharma, E. K. U. Gross, Laser-induced demagnetization at ultrashort time scales: Predictions of TDDFT. *J. Chem. Theory Comput.* **11**, 4870–4874 (2015).

64. M. Krauß, T. Roth, S. Alebrand, D. Steil, M. Cinchetti, M. Aeschlimann, H. C. Schneider, Ultrafast demagnetization of ferromagnetic transition metals: The role of the coulomb interaction. *Phys. Rev. B* **80**, 180407 (2009).

65. N. Jepsen, J. Amato-Grill, I. Dimitrova, W. W. Ho, E. Demler, W. Ketterle, Spin transport in a tunable Heisenberg model realized with ultracold atoms. *Nature* **588**, 403–407 (2020).

66. Y. Tserkovnyak, A. Brataas, G. E. W. Bauer, B. I. Halperin, Nonlocal magnetization dynamics in ferromagnetic heterostructures. *Rev. Mod. Phys.* **77**, 1375–1421 (2005).

67. I. Razdolski, A. Alekhin, U. Martens, D. Bürstel, D. Diesing, M. Münzenberg, U. Bovensiepen, A. Melnikov, Analysis of the time-resolved magneto-optical kerr effect for ultrafast magnetization dynamics in ferromagnetic thin films. *J. Phys. Condens. Matter* **29**, 174002 (2017).

68. M. Battiato, K. Carva, P. M. Oppeneer, Theory of laser-induced ultrafast superdiffusive spin transport in layered heterostructures. *Phys. Rev. B* **86**, 024404 (2012).

69. A. Fognini, T. U. Michlmayr, A. Vaterlaus, Y. Acremann, Laser-induced ultrafast spin current pulses: A thermodynamic approach. *J. Phys. Condens. Matter* **29**, 214002 (2017).

70. L. Braicovich, G. van der Laan, Rationale for femtosecond magnetism explored with x-ray core-hole excitation. *Phys. Rev. B* **78**, 174421 (2008).

71. L. Braicovich, G. Ghiringhelli, A. Tagliaferri, G. van der Laan, E. Annese, N. B. Brookes, Femtosecond dynamics in ferromagnetic metals investigated with soft x-ray resonant emission. *Phys. Rev. Lett.* **95**, 267402 (2005).

72. J. Kondo, Resistance minimum in dilute magnetic alloys. *Prog. Theor. Phys.* **32**, 37–49 (1964).

73. M. A. Ruderman, C. Kittel, Indirect exchange coupling of nuclear magnetic moments by conduction electrons. *Phys. Rev.* **96**, 99–102 (1954).

74. T. Kasuya, A theory of metallic ferro- and antiferromagnetism on Zener's model. *Prog. Theor. Phys.* **16**, 45–57 (1956).

75. K. Yosida, Magnetic properties of Cu-Mn alloys. *Phys. Rev.* **106**, 893–898 (1957).

76. M. Stiles, Interlayer exchange coupling. *J. Magn. Magn. Mater.* **200**, 322–337 (1999).

77. V. Korenev, M. Salewski, I. Akimov, V. Sapega, L. Langer, I. Kalitukha, J. Debus, R. Dzhioev, D. Yakovlev, D. Müller, C. Schröder, H. H. G. Karczewski, M. Wiater, T. Wojtowicz, Y. G. Kusrayev, M. Bayer, Long-range *p–d* exchange interaction in a ferromagnet–semiconductor hybrid structure. *Nat. Phys.* **12**, 85–91 (2016).

78. V. Korenev, I. Kalitukha, I. Akimov, V. Sapega, E. Zhukov, E. Kirstein, O. Ken, D. Kudlacik, G. Karczewski, M. Wiater, T. Wojtowicz, N. Ilyinskaya, N. Lebedeva, T. Komissarova, Y. Kusrayev, D. Yakovlev, M. Bayer, Low voltage control of exchange coupling in a ferromagnet-semiconductor quantum well hybrid structure. *Nat. Commun.* **10**, 2899 (2019).

79. T. Kampfrath, M. Battiato, P. Maldonado, G. Eilers, J. Nötzold, S. Mährlein, V. Zbarsky, F. Freimuth, Y. Mokrousov, S. Blügel, M. Wolf, I. Radu, P. M. Oppeneer, M. Münzenberg, Terahertz spin current pulses controlled by magnetic heterostructures. *Nat. Nanotechnol.* **8**, 256–260 (2013).

80. A. Alekhin, I. Razdolski, N. Ilin, J. P. Meyburg, D. Diesing, V. Roddatis, I. Rungger, M. Stamenova, S. Sanvito, U. Bovensiepen, A. Melnikov, Femtosecond spin current pulses generated by the nonthermal spin-dependent seebeck effect and interacting with ferromagnets in spin valves. *Phys. Rev. Lett.* **119**, 017202 (2017).

81. I. Razdolski, A. Alekhin, N. Ilin, J. P. Meyburg, V. Roddatis, D. Diesing, U. Bovensiepen, A. Melnikov, Nanoscale interface confinement of ultrafast spin transfer torque driving non-uniform spin dynamics. *Nat. Commun.* **8**, 15007 (2017).

82. P. B. Johnson, R. W. Christy, Optical constants of the noble metals. *Phys. Rev. B* **6**, 4370–4379 (1972).

83. A. D. Rakić, Algorithm for the determination of intrinsic optical constants of metal films: Application to aluminum. *Appl. Optics* **34**, 4755–4767 (1995).

84. P. B. Johnson, R. W. Christy, Optical constants of transition metals: Ti, V, Cr, Mn, Fe, Co, Ni, and Pd. *Phys. Rev. B* **9**, 5056–5070 (1974).

85. S. Lee, Y. Chung, Surface characteristics of epitaxially grown Ni layers on Al surfaces: Molecular dynamics simulation. *J. Appl. Phys.* **100**, 074905 (2006).

86. C. Kim, Y. Chung, First-principles calculations of atomistic behaviors in Ni/Al (001) and Al/Ni (001) system. *Jpn. J. Appl. Phys.* **44**, 5700 (2005).

87. A. Hassani, A. Makan, K. Sbiaai, A. Tabyaoui, A. Hasnaoui, Molecular dynamics study of growth and interface structure during aluminum deposition on Ni(1 0 0) substrate. *Appl. Surf. Sci.* **349**, 785–791 (2015).

88. R. Xu, M. Falk, T. Weihs, Interdiffusion of Ni-Al multilayers: A continuum and molecular dynamics study. *J. Appl. Phys.* **114**, 163511 (2013).

89. V. Turlo, O. Politano, F. Baras, Alloying propagation in nanometric Ni/Al multilayers: A molecular dynamics study. *J. Appl. Phys.* **121**, 055304 (2017).

90. M. Cherukara, T. Germann, E. Kober, A. Strachan, Shock loading of granular Ni/Al composites. Part 2: Shock-induced chemistry. *J. Phys. Chem. C* **120**, 6804–6813 (2016).

91. T. Mitsui, S. Sakai, S. Li, T. Ueno, T. Watanuki, Y. Kobayashi, R. Masuda, M. Seto, H. Akai, Magnetic Friedel oscillation at the Fe(001) surface: Direct observation by atomic-layer-resolved synchrotron radiation ^{57}Fe Mössbauer spectroscopy. *Phys. Rev. Lett.* **125**, 236806 (2020).

92. V. Stephanovich, E. Kirichenko, V. Dugaev, J. Barnaś, Dynamic Friedel oscillations on the surface of a topological insulator. *Phys. Rev. B* **105**, 075306 (2022).

93. V. Stephanovich, V. Dugaev, V. Litvinov, J. Berakdar, Ultrafast dynamics of indirect exchange interaction and transient spin current generation in a two-dimensional electron gas. *Phys. Rev. B* **95**, 045307 (2017).

94. G. Zhang, M. Si, T. George, Laser-induced ultrafast demagnetization time and spin moment in ferromagnets: First-principles calculation. *J. Appl. Phys.* **117**, 17D706 (2015).

95. G. Zhang, M. Si, Y. Bai, T. George, Magnetic spin moment reduction in photoexcited ferromagnets through exchange interaction quenching: Beyond the rigid band approximation. *J. Phys. Condens. Matter* **27**, 206003 (2015).

96. Z. Chen, L. Wang, Role of initial magnetic disorder: A time-dependent ab initio study of ultrafast demagnetization mechanisms. *Sci. Adv.* **5**, eaau8000 (2019).
